# A Computational Framework for Studying Gut-Brain Axis in Autism Spectrum Disorder

**DOI:** 10.3389/fphys.2022.760753

**Published:** 2022-03-07

**Authors:** Faiz Khan Mohammad, Meghana Venkata Palukuri, Shruti Shivakumar, Raghunathan Rengaswamy, Swagatika Sahoo

**Affiliations:** ^1^Department of Chemical Engineering, Indian Institute of Technology Madras, Chennai, India; ^2^Initiative for Biological Systems Engineering, Indian Institute of Technology Madras, Chennai, India

**Keywords:** autism spectrum disorder (ASD), gut-brain axis, gut microbiota, constraint-based metabolic modelling (CBM), physiological-based pharmacokinetic modelling (PBPK)

## Abstract

**Introduction:**

The integrity of the intestinal epithelium is crucial for human health and is harmed in autism spectrum disorder (ASD). An aberrant gut microbial composition resulting in gut-derived metabolic toxins was found to damage the intestinal epithelium, jeopardizing tissue integrity. These toxins further reach the brain *via* the gut-brain axis, disrupting the normal function of the brain. A mechanistic understanding of metabolic disturbances in the brain and gut is essential to design effective therapeutics and early intervention to block disease progression. Herein, we present a novel computational framework integrating constraint based tissue specific metabolic (CBM) model and whole-body physiological pharmacokinetics (PBPK) modeling for ASD. Furthermore, the role of gut microbiota, diet, and oxidative stress is analyzed in ASD.

**Methods:**

A representative gut model capturing host-bacteria and bacteria-bacteria interaction was developed using CBM techniques and patient data. Simultaneously, a PBPK model of toxin metabolism was assembled, incorporating multi-scale metabolic information. Furthermore, dynamic flux balance analysis was performed to integrate CBM and PBPK. The effectiveness of a probiotic and dietary intervention to improve autism symptoms was tested on the integrated model.

**Results:**

The model accurately highlighted critical metabolic pathways of the gut and brain that are associated with ASD. These include central carbon, nucleotide, and vitamin metabolism in the host gut, and mitochondrial energy and amino acid metabolisms in the brain. The proposed dietary intervention revealed that a high-fiber diet is more effective than a western diet in reducing toxins produced inside the gut. The addition of probiotic bacteria *Lactobacillus acidophilus*, *Bifidobacterium longum longum*, *Akkermansia muciniphila*, and *Prevotella ruminicola* to the diet restores gut microbiota balance, thereby lowering oxidative stress in the gut and brain.

**Conclusion:**

The proposed computational framework is novel in its applicability, as demonstrated by the determination of the whole-body distribution of ROS toxins and metabolic association in ASD. In addition, it emphasized the potential for developing novel therapeutic strategies to alleviate autism symptoms. Notably, the presented integrated model validates the importance of combining PBPK modeling with COBRA -specific tissue details for understanding disease pathogenesis.

## Introduction

Autism spectrum disorder (ASD) is a complex neurodevelopment disorder. Social impairment, reduced cognitive capability, communication deficits, and stereotyped body behavior are typical clinical characteristics of ASD (Chen et al., 2015). Although genetic and environmental factors have traditionally defined the development of ASD (Chen et al., 2009; Murray et al., 2009; Chaste and Leboyer, 2012), recent studies claim that this is only present in a minority of cases, and that there has been more emphasis on the importance of inherent metabolic disturbances (Frye et al., 2013; Chen et al., 2015). In many cases, comorbidity patterns describe the pathogenesis of autism, most notably gastrointestinal defects and abnormal gut microbiome composition (Adams et al., 2011; Wang et al., 2011; Frye et al., 2013, 2015; Cheng et al., 2017). These factors can be transmitted to the brain *via* the gut-brain axis, resulting in neuronal dysfunction (Ding et al., 2017).

Home to a diverse and dynamic bacterial population, the human gastrointestinal tract is influenced by diseases and homeostasis (Cho and Blaser, 2012). A variety of factors affect the interactions between gut microbiota and the host. One of the most crucial factors of host intestinal epithelial dysfunction is gut microbial disequilibrium (dysbiosis), which contributes to increased toxin production within the Gut (Huang and Sikes, 2014). Toxins produced by dysbiosis, such as propionic acid (Adamberg et al., 2014), lipopolysaccharides (Wang et al., 2019), and most importantly, reactive oxygen species (ROS; Frye et al., 2015), stimulate the production of inflammatory cytokines. As a result, it increases permeability in the gut, allowing toxins into the bloodstream where they can cross the blood-brain barrier and cause symptoms of autism (de Theije et al., 2011; Li et al., 2017). Therefore, it reinforces the concept that any intervention successfully treating gastrointestinal dysfunction may alleviate ASD-related brain symptoms.

Investigating bacterial composition in patients with ASD reveals dysbiosis features, such as increased abundance of *Clostridium*, *Bacteroides*, *Desulfovibrio*, *Ruminococcus*, and *Shigella* (Finegold, 2008; Finegold et al., 2010; Ding et al., 2017; Vuong and Hsiao, 2017) followed by reduced levels of *Bifidobacterium*, *Lactobacillus*, *Prevotella*, and *Akkermansia* (Sandler et al., 2000; Oberhardt et al., 2010; Shaaban et al., 2017; Vuong and Hsiao, 2017). Intestinal epithelium cells absorb nutrients and play an essential role as first-line defense against microbial dysbiosis. Elevated levels of reactive oxygen species (ROS), i.e., hydrogen peroxide (H_2_O_2_) and superoxide (${\text{O}}_{2}^{-1}$), is reported in the autistic gut as a cause of epithelial tissue damage (Chauhan and Chauhan, 2006; Frye and Rossignol, 2011; Ming et al., 2012; Frye et al., 2015). Increased gut permeability, thereby, allows these ROS toxins to be transported into the bloodstream and can further breach the blood-brain barrier, affecting the normal function of the brain. These findings, therefore, link gut microbiome with ASD *via* the microbe-gut-brain axis and strongly support the leaky-gut hypothesis. The leaky gut or intestinal hyper-permeability is the widening of tight junctions in the gut wall, leading to gut epithelial cells losing the ability to discern between molecules passing from the gut to the bloodstream and vice versa.

The first goal of this study is to determine the abnormal level of ROS toxins produced in the autistic gut, including superoxide (SOX) and hydrogen peroxide (H_2_O_2_). To calculate ROS toxins produced by metabolic disturbances in the autistic host gut based on dysbiosis features, we present a multicellular metabolic model of gut microbiota and human small intestine that aids in the investigation of the metabolic perturbation occurring in the ASD host intestine. Furthermore, the proposed gut representative model would simulate complex interactions between the gut microbiome and intestinal cells, and predict metabolic changes as potential intervention strategies to mitigate the dysbiosis associated with autism.

**FIGURE 1:**
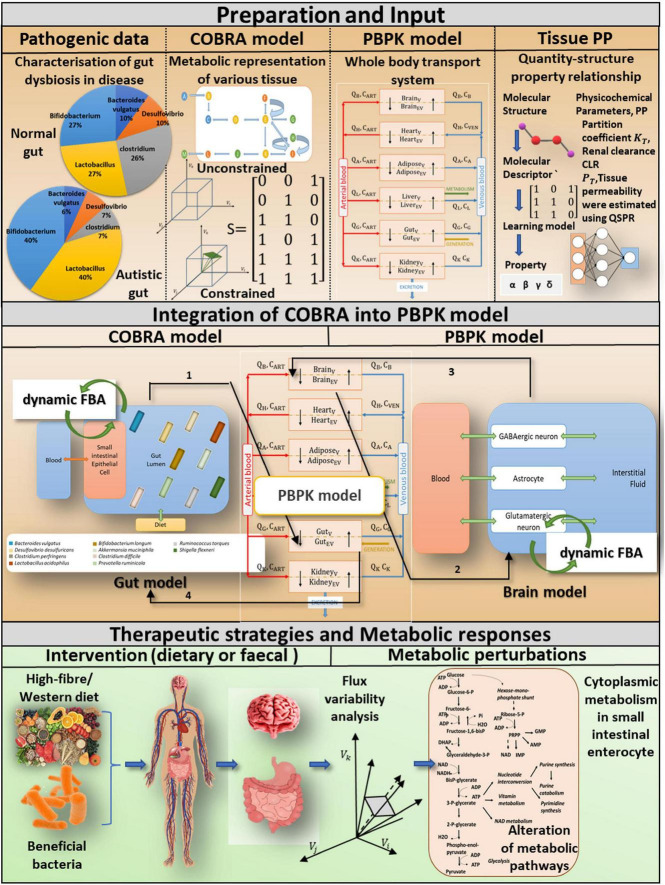
A computational approach for incorporating tissue-specific metabolic information into a whole-body transport model to investigate pivotal role of probiotics and diet in gut dysbiosis-induced diseases. Preparations and input: We basically combine four different levels of information. COBRA is a representative gut model developed by combining patient-specific metagenomic gut dysbiosis traits with omics data from metabolic networks. A comprehensive PBPK model is integrated with the COBRA gut and brain models, and the physicochemical properties of the PBPK model are calculated using the Quantity-structure property relationship QSPR. Integration of COBRA into PBPK model: The new computational framework enables researchers to investigate the metabolic effects of ROS toxins on the host gut and brain. Tissue-specific toxin metabolism is used to quantify the production/consumption of toxins. The toxin reaction rate is constrained in the organ-specific COBRA model by the PBPK-derived toxin distribution. A static version of the dynamic Flux balance analysis algorithm (dynamic FBA) is used to calculate altered flux distributions in the drug perturbed organ-specific COBRA at a given time instant. Therapeutic strategies and metabolic responses: Toxin-induced metabolic perturbations, which result in altered intracellular and extracellular reaction rates, can be predicted using a composite representation of whole-body toxin metabolism. The integrated model explicitly considers specific dietary and probiotic interventions, as well as their impact on autistic symptoms.

Metabolic studies and analyses of elevated concentrations of oxidizable molecules in nervous tissue suggest that proteins involved in normal brain function are susceptible to oxidative modifications, which alter their activity. Specifically, glutamic acid decarboxylase (GAD), an enzyme that converts glutamate to γ-aminobutyric acid (GABA), is vulnerable to oxidative stress (Rojas, 2014; Wasilewska and Klukowski, 2015; Thursby and Juge, 2017). Furthermore, reduced GABA and elevated extracellular glutamate are known for increasing excitotoxicity, which is reported on individuals with ASD (Kern and Jones, 2006; El-Ansary and Al-Ayadhi, 2014; Bjørklund et al., 2020). Excitotoxicity is also linked to oxidative stress. Thus, our second goal is to construct a combined neuronal model representing the ASD brain. This model would help us calculate the level of oxidative stress and resulting GABA and glutamate in the brain and its relationship with oxidative factors arising from gut dysbiosis (Figure 1).

**FIGURE 2:**
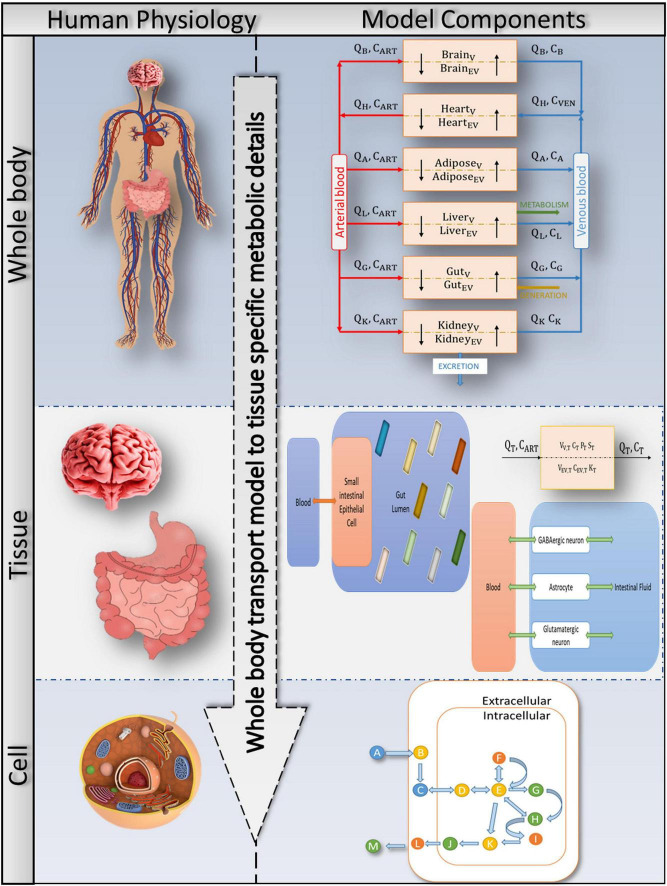
Bringing together whole-body physiology and model components: each tissue of interest is modelled as a two-compartment, permeability-limited, well-stirred tank with vascular and extravascular compartments. The circulatory system connects the various compartments. The cellular biochemistry in the interstitial (extracellular) and intracellular (cytosol, mitochondria, peroxisome, and so on) areas is described using genome-specific COBRA models. A combined PBPK-GSMN model connects tissue-specific metabolic details into a comprehensive representation of a whole-body physiologically based pharmacokinetic (PBPK) model via the shared exchange toxins metabolism.

Toxin generation and exposure to various tissues are challenging to quantify in clinical practice (MacFabe et al., 2007; de Theije et al., 2011; MacFabe, 2012). Furthermore, determination of toxin-induced neuronal abnormalities using just a bacterial community encompassed a gut model (Srikantha and Mohajeri, 2019). As a result, a functional assessment of such metabolic dysfunction inevitably necessitates a computational workflow that connects metabolic disturbance in the host gastrointestinal tract to the brain *via* a whole-body transport system to understand gut dysbiosis in the host and plan therapeutic strategies (Figure 1). In multi-compartment PBPK models, where compartments correspond to different organs of the body, a system of differential equations describes substance concentration (Menzel, 1987; Zhuang, 2016). On the other hand, constraint-based reconstruction and analysis (COBRA; Heirendt et al., 2019) enables the analysis of metabolic perturbations and quantitative prediction of possible physicochemical and biochemically phenotypic states by providing metabolic insight (Figure 1). Thus, the third goal of this research is to create a computational framework that will allow researchers to investigate ROS toxin-induced metabolic perturbations in the host intestine and brain using an effective integration platform (i.e., the whole-body transport PBPK model and tissue-specific COBRA models), as shown in Figure 2. Moreover, integrating the two will provide a comprehensive analysis of symptoms of autism in the gut and their influence on the distant organ brain.

Specifically, the presented framework helps us understand and address the following questions:

1. What are the fundamental biochemical factors that drive autism?
2. What is the role of bacteria-derived toxins/metabolites in autism?
3. Can modeling of host-microbe interactions under different dietary conditions hint toward dietary treatment options for autism?
4. How do species added to the microbiome consortia reveal essential metabolic interactions between gut microbes?

## Materials and Methods

**FIGURE 3:**
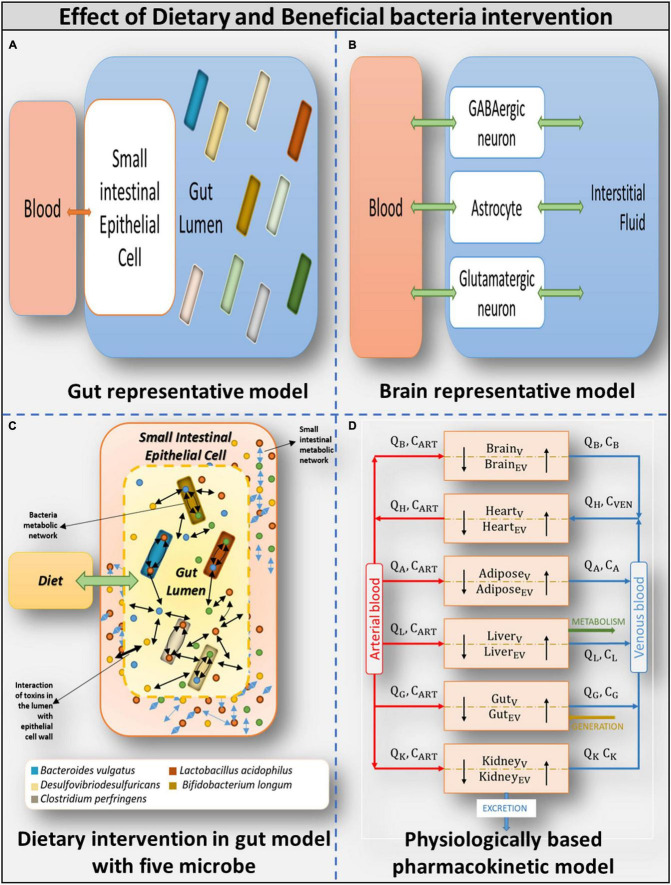
Overview of models used **(A)** Gut representative model: Overall integrated Gut representative model. **(B)** Brain representative model: Brain model and its components. **(C)** Dietary intervention in gut model with five model: Gut representative model interacting with diet and host-microbe and microbe-microbe interaction. **(D)** Physiologically based pharmacokinetic model: Whole-body PBPK model.

To investigate dysbiosis-induced metabolic anomalies in the autistic gut vs. the healthy gut, we employed metagenomic data on bacterial abundance to develop an autism gut representation as a dysbiosis feature (Figure 3). To investigate the effects of ROS exposure on diverse organs, including the brain, we integrated tissue-specific metabolic models with a whole-body physiological-based pharmacokinetic (PBPK) model. Mutual exchange of toxins connects the two modeling methodologies, resulting in an integrated COBRA-PBPK model. Furthermore, genome-scale flow patterns characterizing the intercellular and extracellular reactions in the face of toxin exposure are examined for various amounts of food and bacterial intervention, as well as gut bacterial makeup. Genome-scale flow patterns characterizing intercellular and extracellular reactions in the face of toxin exposure are investigated for a variety of gut bacterial compositions and dietary limitations, with each indicating distinct amount of dietary and bacterial intervention. The outline of framework is shown in Figure 1.

The structure of the framework stands on three essential components:

1. A genome-scale bacterial community model merged with an intestinal epithelial cell model of the host representing the human gut.
2. Combining neuronal models to represent the human brain.
3. The whole-body PBPK transport model considers six organs interacting through the circulatory system of the body.

### Model Construction

#### Personalized Bacterial Community Combined With Host Intestinal Cells to Represent the Human Gut

The human gut microbiome is a diverse, complex system that responds dynamically to its microenvironment. The microbiome has a significant impact on the well-being of humans. Understanding the inherent ability of the human microbiome to interact in both one-way (commensal) and two-way (mutualistic) manners (Zheng et al., 2020), we built a personalized bacterial community in which the gut microbiome is simplified to the point where the pathophysiological condition associated with ASD can be easily captured (Elzinga et al., 2019). The microbial community is also merged with an intestinal host to represent the typical human gut (Figure 3). The microbial community is designed to crosstalk with each other and interact with the host (Heinken and Thiele, 2015; Magnúsdóttir and Thiele, 2018). The bacterial community is built using the Microbiome Modeling Toolbox (Baldini et al., 2018). Individual microbial models were combined in this community modeling design setup by adding reactions to each microbial model, i.e., transporting metabolites from the extracellular space to the common lumen compartment. Metabolites in the lumen compartment are constrained with diet or fecal compartment, enabling bilateral interaction with the environment.

One of the most popular biology methods for studying biological cell systems with mechanistic details is constraint-based reconstruction and analysis (COBRA; Heirendt et al., 2019). Numerous studies have used COBRA tissue modelling to investigate the interaction between gut bacteria and the host gut. The assembly of gut organisms through reconstruction and analysis (AGORA) project (Magnúsdóttir et al., 2016) presents genome-scale metabolic models derived from human gut metagenomic data that can be used to understand how microbial communities influence human metabolism and health. A collection of 773 metabolic reconstructions/models of 13 most common human gut microbe species has been made public (Magnúsdóttir et al., 2016; Bauer and Thiele, 2018; Noronha et al., 2018). Thus, combining an individual constraint-based genome-scale metabolic model of human small intestinal enterocyte cell (sIEC; Sahoo and Thiele, 2013) and gut bacteria implicated in autism will result in a gut metabolic model, as shown in Table 1.

**TABLE 1:** Gut bacterial abundance in autistic compared to normal human gut.

Bacteria	Abundance in the autistic gut compared to normal human gut	References
Bacteroides vulgatus ATCC	Increased	Finegold, 2008; Finegold et al., 2010; Ding et al., 2017; Vuong and Hsiao, 2017
Desulfovibrio desulfuricans subsp. desulfuricans	Increased	Finegold, 2008; Finegold et al., 2010; Ding et al., 2017; Vuong and Hsiao, 2017
Clostridium perfringens	Increased	Finegold, 2008; Finegold et al., 2010; Ding et al., 2017; Vuong and Hsiao, 2017
Lactobacillus acidophilus	Decreased	Sandler et al., 2000; Shaaban et al., 2017; Vuong and Hsiao, 2017
Bifidobacterium longum longum	Decreased	Sandler et al., 2000; Shaaban et al., 2017; Vuong and Hsiao, 2017
Akkermansia muciniphila	Decreased	Sandler et al., 2000; Shaaban et al., 2017; Vuong and Hsiao, 2017
Clostridium difficile	Increased	Sandler et al., 2000; Shaaban et al., 2017; Vuong and Hsiao, 2017
Prevotellaruminicola	Decreased	Sandler et al., 2000; Shaaban et al., 2017; Vuong and Hsiao, 2017
Ruminococcus torques	Increased	Finegold, 2008; Finegold et al., 2010; Ding et al., 2017; Vuong and Hsiao, 2017
Shigella flexneri	Increased	Finegold, 2008; Finegold et al., 2010; Ding et al., 2017; Vuong and Hsiao, 2017

#### Gut Microbiota Representative Models

##### Five Bacteria Personalized Community

A COBRA model of five important bacteria species, *Bacteroides vulgatus*, *Clostridium perfringens*, *Desulfovibrio desulfuricans*, *Lactobacillus acidophilus*, and *Bifidobacterium longum*, shown in Supplementary Files/Additional File 1/Table S11A, was coupled with a genome-scale model of the sIEC, representing the human gut with five models of bacteria (specification of combined model shown in Supplementary Files/Additional File 1/Table S11A. Furthermore, harmful bacteria in higher abundance (Table 1) were connected to represent purely autistic gut, while beneficial bacteria are linked to represent beneficial gut.

The six separate models listed below were developed to assess the impact of dietary intervention.

1. Gut microbiome: high fiber: bacterial community model constrained with high-fiber diet.
2. Gut microbiome: Western: bacterial community model constrained with the Western diet.
3. Gut microbiome harmful: high fiber: harmful bacteria (only bacteria present in abundance in autistic gut) model constrained with high-fiber diet.
4. Gut microbiome harmful: Western: harmful bacteria (only bacteria present in abundance in autistic gut) model constrained with the Western diet.
5. Gut microbiome beneficial: high fiber: Beneficial bacteria (Gut bacteria present in very less percentage in autistic gut) model constrained with High fiber diet.
6. Gut microbiome Beneficial: Western: beneficial bacteria community (gut bacteria present in very less percentage in autistic Gut) model constrained with the Western diet.

The sIEC model, hs_sIEC61 (Sahoo and Thiele, 2013), was expanded with new pathways to capture the metabolism of bacteria-derived toxin metabolites. A total of 12 reactions and seven metabolites were added to include the metabolism of propionic acid and essential reactive oxygen species, such as H_2_O_2_ and SOX (Supplementary Table/Additional File 1/Table S12). The gut bacteria and sIEC interact through exchange of metabolites *via* the lumen compartment. Thus, to generate the combined model, 665 luminal transport reactions were added for extracellular metabolites of the bacterium models (Supplementary Files/Table 1).

For each of the gut representative models, exchange reactions were constrained by intestinal conditions. An example is the aerobic condition of the small intestine, by constraining the lower bound of oxygen exchange reactions in bacterial models to lb = −1 mmol/gDW/hr (Supplementary Files/Table 1).

##### Ten Bacteria Personalized Community

To understand the scalability factor in our personalized gut model, we increased the size of the community to ten bacteria (both harmful and beneficial). Supplementary Files/Additional File 1/Table S11B shows the ten selected bacteria in the expanded ten microbial communities and their specifications in individual and combined models. In addition, the entire technique for building the model is detailed in Supplementary Files/Additional File 1.

#### Combined Neuronal Model Representing the Human Brain

Recent investigations have shown that increased levels of oxidative stress in the brain result in increased level of glutamate and reduced level of GABA neurotransmitters (Coghlan et al., 2012). We built a brain model by combining previously published GABA and glutamate neuronal models (Lewis et al., 2010). Each neuronal model comprises two cell types, the neuron and the supporting astrocyte cell (Supplementary Files/Additional File 1/Table S11A). After quality checks and corrections, the individual neuronal models were then combined to form a combined whole-brain model (Supplementary Files/Table 1). Furthermore, Supplementary Files/Additional File 1/Table S11A shows the published neuronal model specifications and the whole-brain model. Details of the model components are shown in Supplementary Files/Table 1.

#### Whole-Body Physiological-Based Pharmacokinetic Transport Model

Constraint-based reconstruction and analysis operates under steady-state conditions (Heirendt et al., 2019). Hence, to capture the dynamics of autism pathogenesis, a whole-body physiological-based pharmacokinetic (PBPK) modeling approach is required (Bois, 2010), precisely to model the transport and effect of gut-derived toxins (H_2_O_2_*and*O^−2^) through the gut-brain-axis in the brain in autism. Therefore, the third most crucial component in building the computational framework is the whole-body transport model. We considered a semi-detailed PBPK model of six organs, as shown in Figure 3.

The PBPK transport model considers each tissue type of interest as a two-compartment, permeability-limited, well-stirred tank consisting of a vascular compartment and an extravascular compartment (Thompson and Beard, 2012; shown in Figure 3). The various compartments communicate with one another *via* the circulatory system of the body. Thus, movement and accumulation of the compound as a function of time were quantified in each tissue type through a set of ordinary differential equations (shown in Supplementary Files/Additional File 1/Table S1). The steps involved in building a PBPK model is shown in Supplementary Files/Additional File 1/Table S2, and details are shown in Supplementary Files/Additional File 1/Table S2.

One of the main objectives of this study is to examine the effect of ROS in the brain of individuals with autism; therefore, we included tissues that metabolize ROS, specifically SOX and H_2_O_2_, or whose function is affected by ROS in the full-body PBPK model. Thus, the minimalistic PBPK model included (i) the heart as a source and sink of the circulatory system, (ii) gut, and (iii) brain, as they were the central organs in our study. Since increased ROS levels are implicated in adipocyte dysfunction (Castro et al., 2016), (iv) adipose tissue was included in the model. Being the central metabolic organ, (v) the liver was included to maintain the generality of the model, and (vi) the kidneys were included to allow for excretion shown in components of the model in Figure 3. To formulate the model equations, tissue-specific characteristics that affect the uptake of the molecule of interest needed to be defined.

As mentioned earlier, elevated levels of ROS in the autistic gut leads to inflammation of the gut epithelial wall, which further results in increased permeability of the gut wall. It was also determined that once toxins pass through the blood-brain barrier, they may cause brain inflammation (de Theije et al., 2011). Thus, to account for the increased epithelial permeability of the gut wall and brain of individuals with autism vs. controls, a tissue model that accounts for organ permeability in its formulation was used. Thus, permeability-limited, two sub-compartment models were formulated and used for further analysis (Figure 3).

Governing mass balance equations for the permeability-limited model are as follows:


(1) $$\frac{{\text{fV}}_{\text{T}}}{1+\text{f}}\,\frac{{\text{dC}}_{\text{T}}}{\text{dt}}={\text{Q}}_{\text{T}}\,{\text{C}}_{\text{ART}}-{\text{Q}}_{\text{T}}\,{\text{C}}_{\text{T}}+\frac{{\text{P}}_{\text{T}}\,{\text{S}}_{\text{T}}\,{\text{C}}_{\text{EV},\text{T}}}{{\text{K}}_{\text{T}}}-{\text{P}}_{\text{T}}\,{\text{S}}_{\text{T}}\,{\text{C}}_{\text{T}}$$

(2) $$\frac{{\text{V}}_{\text{T}}}{1+\text{f}}\,\frac{{\text{dC}}_{\text{EV},\text{T}}}{\text{dt}}={\text{P}}_{\text{T}}\,{\text{S}}_{\text{T}}\,{\text{C}}_{\text{T}}-\frac{{\text{P}}_{\text{T}}\,{\text{S}}_{\text{T}}\,{\text{C}}_{\text{EV},\text{T}}}{{\text{K}}_{\text{T}}}$$

(3) $$\text{f}=\frac{{\text{V}}_{\text{T}}}{{\text{V}}_{\text{EVT}}}$$

The final set of ordinary differential equations used was simply the above two mass balance equations written for each of the six tissue types, i.e., brain, heart, adipose, liver, gut, and kidney (Supplementary Files/Additional File 1/Table S1), with additional generation and consumption terms obtained from the COBRA models, as detailed in the supporting information text.

### Parameter Estimation in the Physiological-Based Pharmacokinetic Model

The novelty of the PBPK model lies in its semi-detailed and highly curated nature, wherein the physiological and biochemical parameterization of the model was primarily literature-driven (Supplementary Files/Additional File 1/Table 2D). While physiological parameters like blood flow rate through different tissue types, tissue surface area, and tissue volume, and biochemical parameters like enzymatic reaction rate and Michaelis constant were largely obtained from the literature, physicochemical parameters like partition coefficient *K*_*T*_, renal clearance *CL*_*R*_, and tissue permeability *P*_*T*_, were estimated using Quantitative structure-property relationship (QSPR; Supplementary Files/Additional File 1/Table S3).

### Quantitative Structure-Property Relationship

Quantitative structure-property relationship (QSPR; Katritzky et al., 2005; Roy et al., 2015) models assume a strong correlation between the structure of a compound and its physical and chemical properties like melting point, boiling point, and in our case, tissue-plasma partition coefficient. The fundamental assumption behind QSPR is that molecules with similar structures have similar observable properties, and that molecules with different structures have different observable properties. QSPR models are built with predictors consisting of theoretical molecular descriptors and fragment descriptors of a compound, derived solely from the molecular structure of the compound and not from experimental data (Liu et al., 2005). The output variable is the physicochemical property of interest. Further information on calculation of physicochemical properties is discussed in Supplementary Files/Additional File 1.

### Integration of Tissue-Specific Constraint-Based Metabolic Model and a Whole-Body Physiological-Based Pharmacokinetic Transport Model

Integrated modeling of the gut-brain link in autism pathogenesis combines the following components, i.e., representative gut community model and combined neuronal model with the whole-body dynamic PBPK model (Figures 1, 2). Integration is achieved by stepwise discretization into time steps. In each time step, the maximum consumption or production of toxin metabolites by the COBRA model (modeled as input and output exchange reactions) was calculated, and fluxes through these reactions were extracted and converted to a concentration value that is fed in the PBPK model to calculate the constraint for the next time step in the brain and gut specific-genome scale metabolic model. An initial concentration of 0.0001 M was assumed for H_2_O_2_ and SOX, and a step size of 15 min was used for the integration and simulation ran for 6 h.

In the integrated model, concentrations were changed in every time step not only because of consumption or production of metabolites by the COBRA model (modeled as input and output reactions, respectively) (Supplementary Files/Additional File 1/Tables S12, S13) but also because of the rate of metabolite transport. The transport depends on permeability and other parameters taken into consideration by the PBPK model, achieved by computing bounds on the input fluxes to the COBRA model using the concentrations obtained from the PBPK model in every time instant. To ensure metabolite input into the COBRA model, the reversible exchange reaction was replaced by two irreversible reactions, an input reaction constrained by the PBPK model bound and an output reaction, as shown in Supplementary Files/Additional File 1/ Table S13.

**FIGURE 4:**
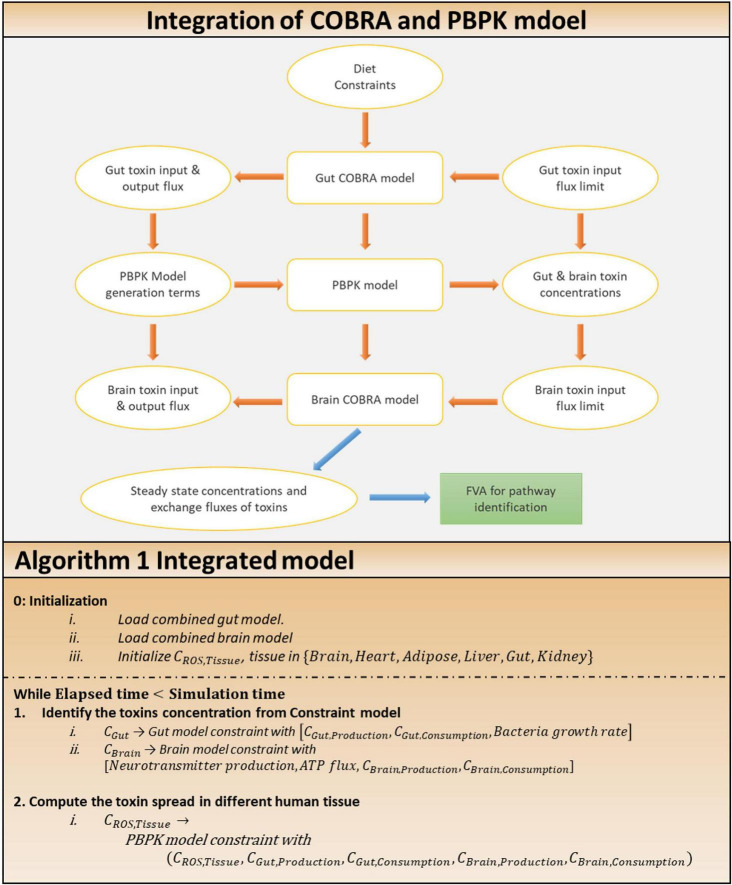
Approach for the integration of COBRA and PBPK model. Integration of COBRA and PBPK model: the integrated model, showing the interactions between the gut, brain COBRA models and the transport PBPK model. Simulation of the integrated model yields the steady state exchange fluxes of toxins used for further analysis through FVA to identify altered pathways. Algorithm 1: algorithm for the integration of two model.

The COBRA analysis calculated its consumption and generation terms, which were then used as the metabolite’s actual input and output exchange fluxes by the PBPK model. Furthermore, the PBPK approach combines concentrations for the next instant. The process was repeated with the obtained concentrations. The entire procedure is described in Algorithm 1 (Figure 4).

The following is a simulation of the representative gut COBRA model for toxin calculation. Under the maximal bounds set for the toxin inputs, pareto-optimal growth (discussed in the section “Model Analysis” below) of all cell types in the model was computed and set as bounds for the respective biomass reactions. Then, maximal toxin production (output exchange) fluxes were determined by pareto-optimization and set as bounds to the output exchange constraints. The maximal toxin input fluxes were determined as consumption terms for the PBPK model by pareto-optimization, and output exchange fluxes as generation terms. The COBRA simulation used the same procedure for the brain in each time step, except that ATP maintenance reaction was maximized rather than biomass_maintenance, as shown in Supplementary Files/Additional File 1/Table S14. Furthermore, neurotransmitter production fluxes (modeled as demand reactions for GABA and glutamate) were computed by pareto-optimization under the calculated constraints. The steps involved in integrated model analysis are shown in Figure 4.

As shown in Supplementary Files/Additional File 1/Table S15, for each time step, the following pareto-optimization problem is solved depending on the net objective:


(4) $$\max\,w^{\text{T}}\,\bar{\text{v}}$$

$$\bar{\text{v}}\in \text{X}\,\left(\mathrm{paretofeasible}\,\mathrm{space}\right)$$

$$\mathrm{such}\,\mathrm{that},S*v_{i}=0$$

$$\text{lb}\leq {\text{v}}_{\text{i}}\leq \text{ub}$$

Unlike the COBRA model, a flux-based model, the PBPK model requires toxins to be input in concentration. To establish a link between production and consumption of toxins in two platforms of dynamic and steady-state modeling, the flux from COBRA is appropriately converted to concentration using Equation 5:


$$\text{Flux}\,\left(\frac{\text{nmols}}{\mathrm{gram}\,\mathrm{weight}\,\mathrm{of}\,\mathrm{tissue}\cdot \text{hr}}\right)$$

(5) $$=\text{C}\,\left(\text{M}\right)\times \frac{\mathrm{volume}\,\mathrm{of}\,\mathrm{extravascular}\,\mathrm{region}\,\mathrm{of}\,\mathrm{tissue}\,\mathrm{in}\,\mathrm{rat}}{\mathrm{gram}\,\mathrm{weight}\,\mathrm{of}\,\mathrm{organ}\,\mathrm{in}\,\mathrm{rat}}$$

The volume of the extravascular region of the brain and the gut was obtained from the PBPK model, and gram weight was obtained from experimental values defined for rats, i.e., the small intestine was 7.26 g (Sheldon and Robinson, 2007), and the brain was 2 g (Miller, 1971; Zoroglu et al., 2004).

### Model Analysis

Having constructed a novel computational setup for integrating different model types, we now present the framework for its analysis.

#### Analyzing Dietary Intervention

An integrated model is developed in this study to understand the complex interactions between gut microbiome composition and human host under different dietary constraints in the autistic vs. healthy case. The COBRA genome-scale metabolic models of the gut and brain were used to represent the human host. To transport gut-derived metabolites across the COBRA genome-scale metabolic models of the tissues, the PBPK model was used. Two different dietary regimes were used as diet constraints, i.e., a high-fiber and a Western diet (details in Supplementary Files/Additional File 1). The combined gut model was constrained with diet by only opening exchanges in the model that correspond to nutrients in the diet for uptake. Six separate models are constructed and analyzed for the effect of dietary intervention.

Furthermore, in constraint-based models, the PBPK model aids in establishing precise bounds for toxin exchange reactions. The integrated approach is essential, because in the absence of a PBPK model, constraint-based models would have been initialized with arbitrary bounds not representing actual physiological conditions. We demonstrated this by choosing the steady-state flux values to be 100 times the actual values obtained from the PBPK model to simultaneously study the effect of increase in toxin fluxes (Supplementary Files/Table 2).

#### Analyzing the Effect of Varying Gut Bacteria Compositions on Autistic Gut

The abnormal composition of gut bacteria inside the human gut, a source of gut-brain-axis inflammation associated with autism, can be significantly reduced by intervention with beneficial bacteria. To illustrate the intervention with beneficial bacteria, we developed five separate models of the gut microbiome to analyze the effect of varying compositions of gut bacteria, representing their different levels in autism, as shown below.

Five separate models of the gut microbiome to analyze the effect of varying gut bacteria composition, representative of their different levels in autism are listed below:

1. Purely autistic gut (also gut microbiome-harmful): Constructed by combining sIEC and the individual models of harmful bacteria grown at the maximum rate.
2. Typical autistic gut: Constructed by combining the sIEC model with the individual models of all the five bacteria, with the harmful bacteria forming 80% of the total gut bacterial population.
3. Corrected cut (also gut microbiome): Constructed by combining the sIEC model with the individual models of all the five bacteria grown at the maximum rate, representing a scenario where symptoms of autism are alleviated by administering probiotics.
4. Typical healthy gut: Constructed by combining sIEC and models of all the five bacteria, with the beneficial bacteria forming 80% of the total gut bacterial population.
5. Purely healthy gut (also gut microbiome-beneficial): Constructed by combining the SIEC model with the models of beneficial bacteria. Components of the gut models are illustrated in Figure 3, and model details are specified in Supplementary Files/Additional File 1.

Furthermore, appropriate weights are given to two classes of bacteria to show different levels of beneficial bacteria intervention (as shown in Table 2A).

**TABLE 2:** Details of the microbiome community models.

(A) Weights for the biomass reaction at different percentage of beneficial bacteria abundance							
**Five microbial community model**							
**S. No.**	**Beneficial bacteria %**	**Fraction contribution of the biomass reaction to the final objective function in the weighted Pareto-optimality**					
		**LA**	**BL**	**BV**	**DD**	**CP**	**Biomass SIEC**
1	20	0.0833	0.0833	0.2222	0.2222	0.2222	0.1667
2	40	0.1667	0.1667	0.1667	0.1667	0.1667	0.1667
3	60	0.25	0.25	0.1111	0.1111	0.1111	0.1667
4	80	0.3333	0.3333	0.0556	0.0555	0.0556	0.1667

**(B) Growth rates of gut microbiome cells (mmol/gDW/hr)**							
**Five microbial community: Individual and combined model specification**							
**Model**	**Diet**	**BV**	**DD**	**CP**	**LA**	**BL**	**SIEC**

*Gut microbiome*	*Western*	1.06472	1.0585	0.3129	0.7336	0.9927	0.00972
	*High-Fiber*	1.05109	1.04102	0.3057	0.73197	1.0106	0.00933
*Gut microbiome – Harmful*	*Western*	1.3402	1.2940	0.4529	–	–	0.01601
	*High-Fiber*	1.2675	1.2220	0.4448	–	–	0.01601
*Gut microbiome – Beneficial*	*Western*	–	–	–	0.1586	0.1511	0.01984
	*High-Fiber*	–	–	–	0.3098	0.6589	0.02546

#### Multi-Objective Optimization of Growth Rates or Adenosine Triphosphate Maintenance Fluxes of Individual Cells

Multi-objective optimization techniques are required to determine the growth rates or ATP maintenance fluxes of individual cells in a multicellular model, such as the gut microbiome and brain (Nagrath et al., 2007). Other multi-objective optimization problems on which our research focuses, such as production and consumption of various toxins and neurotransmitters, were also raised, and each compound was given equal importance. Such situations were encountered while simulating the typical autistic gut and typical healthy gut models.

##### Pareto-Optimization Solution to the Multi-Objective Optimization Problem

**FIGURE 5:**
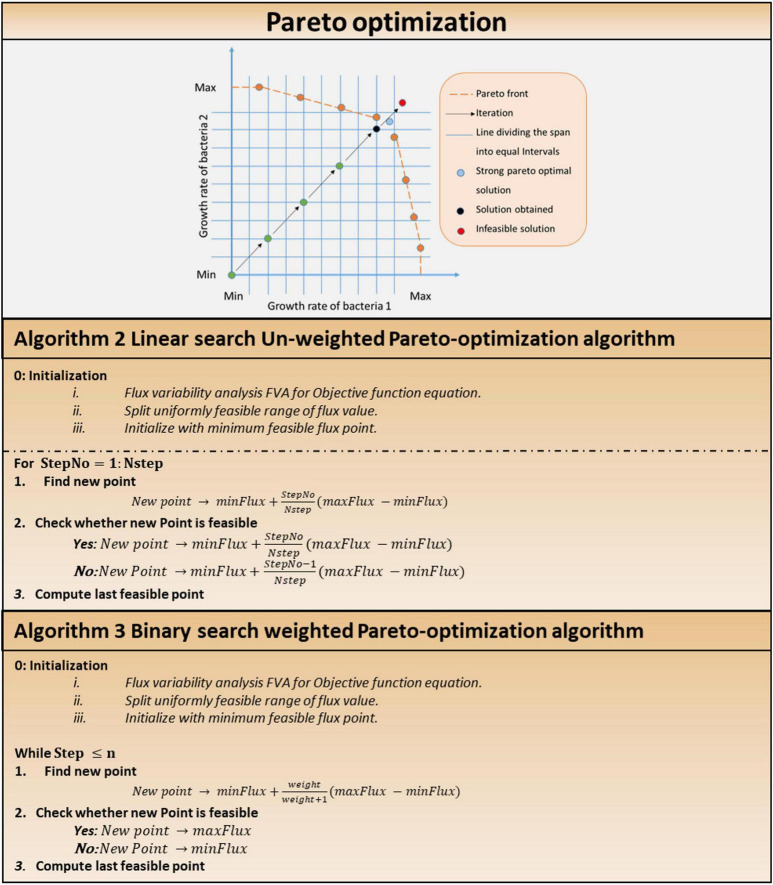
Multi-objective optimization in multi-cellular system. Pareto optimization: pareto optimization search technique. Algorithm 2: linear Search unweighted Pareto optimization algorithm. Algorithm 3: binary Search weighted Pareto-optimization algorithm.

Pareto-optimal solution is required for multi-objective optimization. Two pareto-optimization algorithms were developed and implemented, employing linear search (Algorithm 2, Figure 5) for equally weighted pareto-optimization and binary search for unequally weighted pareto-optimization. Each objective (e.g., the biomass of each cell) was assigned an equal weighting in a system with an equal number of cells of each type, because it is expected that each cell in the system will try to maximize its growth regardless of external conditions.

##### Linear Search

In a system with equal number of cells of each type, equal weightages are awarded to each objective (say, biomass of each cell), since it is expected that each cell in the system will try to maximize its own growth disregarding external conditions (i.e., a cell tries to survive in any environment and does not give more importance to the survival of other cells over its own).

##### Weighted Pareto-Optimization

Other pareto-optimal solutions can be obtained by choosing different slopes of lines from the starting point (minimum fluxes in each dimension). Consider the following scenario: in the system, bacterium 1 accounts for 80% of the total microbial composition, while bacterium 2 accounts for remaining 20%. The algorithm will assign weights to the increment step at a 1:4 ratio. Figure 5 depicts a graphical representation of Algorithm 1 in the solution search space. Stopping criteria include maximum number of iterations or the Euclidean norm of the difference among subsequent iterates falling below a specified tolerance value. In weighted pareto-optimization, a binary search technique finds other pareto-optimal solutions by varying the slopes of lines from the starting point (minimum fluxes in each dimension).

#### Crosstalk Between the Gut Microbiome and Contribution to Secretion Products

The gut microbiome model enables the investigation of microbial interaction, which is determined by performing flux variability analysis on the gut microbiome model and identifying active reactions that were revealed. Flux variability analysis (FVA) (Gudmundsson and Thiele, 2010) is helpful to determine the minimum and maximum fluxes for responses in a network while maintaining some network state, such as the rate of biomass production. With the powerful FVA tool in hand, new questions about the flexibility of biochemical reactions in the network of various organism phenotype states can be addressed.

#### Model Comparison for Analyzing the Metabolic Response

Flux variability analysis, i.e., FVA, which computes network variability, is performed to analyze metabolic perturbation in the brain and gut model under varying simulation constraints. We defined two metrics using the fluxes of a reaction in a model. Equations 6 and 7 are used to compare the responses of a model to the effect of change in conditions.


(6) $$\mathrm{Mean}\,\mathrm{shift}=\left|\frac{{\text{FVA}}_{\text{Max}\,1}+{\text{FVA}}_{\text{Min}\,1}}{2}-\frac{{\text{FVA}}_{\text{max}\,2}+{\text{FVA}}_{\text{min}\,2}}{2}\right|$$

(7) $$\mathrm{Range}\,\mathrm{change}=\left({\text{FVA}}_{\text{Max}\,1}-{\text{FVA}}_{\text{Min}\,1}\right)-\left({\text{FVA}}_{\text{Max}\,2}-{\text{FVA}}_{\text{Min}\,2}\right)$$

Before changing conditions, *FVA*_*Max1*_ and *FVA*_*Min1*_ were the maximum and minimum fluxes of a reaction in a model. *FVA*_*Max2*_ and *FVA*_*Min2*_ are the maximum and minimum fluxes after the conditions are changed.

Both these metrics were computed for each of the reactions of interest in the model, and the reactions were ordered in decreasing order of the mean shift. In case of a tie, the decreasing order of range change was used to determine the order. Thus, an order was determined for the effect of modifications in a model on all the reactions of interest.

Moreover, a topological analysis, called pathway analysis, module measures metabolic subsystems in a metabolic network that have a significant shift in the flux of reactions associated with the subsystems. To identify statistically significant subsystems having significant pathway shifts, we used pathway score as defined in Equation 8. Subsystems with higher pathway score represents high degree of changes in the reaction.


(8) $$\mathrm{Pathway}\,\mathrm{score}=\frac{\text{No}.\mathrm{of}\,\mathrm{shifted}\,\mathrm{reactions}}{\mathrm{Total}\,\mathrm{no}.\mathrm{of}\,\mathrm{reactions}\,\mathrm{in}\,\mathrm{subsystem}}$$

#### Metabolic Pathways Response Due to Increased Oxidative Stress

The effect of oxidative stress was assessed by comparing the toxic and non-toxic models (detailed in the section “Results”) for both the brain and Gut by FVA, and was determined by setting the lower bounds of toxin input and output exchanges to the steady-state flux values obtained from the integrated model. The non-toxic model allowed for lower exchanges of toxins. Implications of the leaky-gut hypothesis and increased permeability of the blood-brain barrier were examined through simulation of this phenomenon on the brain model by allowing toxins into the brain model.

Furthermore, the effect of toxins on the brain was studied by comparing a toxic with a non-toxic model of the brain. The integrated model’s steady-state values for brain toxin exchanges were used to set the toxin exchange bounds for the brain model. Likewise, for the gut model, FVA was performed to compare the models in both analyses.

## Results

### Dietary Influence on Gut Bacterial Growth

**FIGURE 6:**
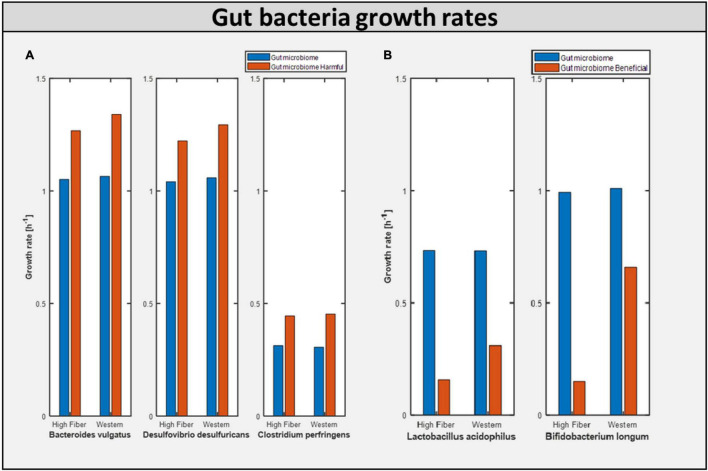
Bacterial growth rate in differential model. **(A)** Growth of harmful bacteria as compared in the two model Gut microbiome and Gut microbiome harmful. **(B)** Growth of beneficial bacteria as compared in the two model Gut microbiome and Gut microbiome beneficial.

As mentioned earlier, one of the major objectives of this study was to determine the impact of a potential dietary intervention strategy on autism. A Western diet and a high-fiber diet were selected as two typical dietary patterns that promote growth of beneficial bacteria. Each of the aforementioned gut representative models, both autistic (gut microbiome-harmful) and healthy (gut microbiome), was subjected to dietary and beneficial bacteria interactions. We observed that the addition of the Western diet to the purely autistic gut, i.e., gut microbiome-harmful, decreased the growth rates of each of the harmful bacteria compared to the gut microbiome model, despite having lesser quantity than the harmful bacteria, as shown in Figure 6; growth rates are shown in Table 2B. Since the growth rate of healthy bacteria increased (when compared to the Western diet) to a larger extent compared to that of harmful bacteria in the high-fiber diet, especially with higher percentage of healthy bacteria in the gut, we can infer that the high-fiber diet is better than the Western diet for growth of beneficial bacteria, and consequently, alleviation of autism symptoms, as discussed below.

### Toxins Derived in the Gut Microbiome Model

**FIGURE 7:**
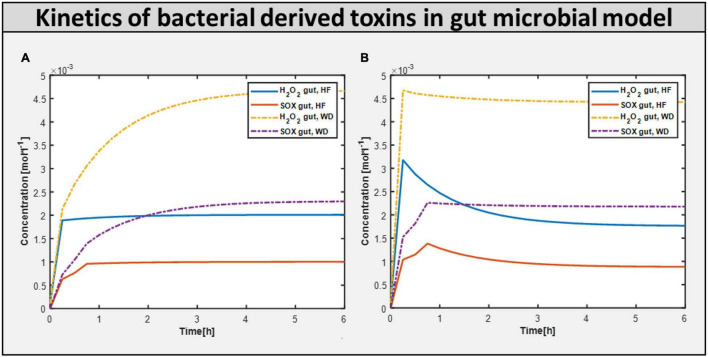
Bacteria derived toxins. **(A)** Toxins produced H_2_O_2_ (hydrogen perxoide) and SOX(superoxide) in Gut microbiome harmful model constrained with differential diet. **(B)** Toxins produced H_2_O_2_ and SOX in Gut microbiome beneficial model constrained with differential diet High fiber and Western.

Calculating the toxins produced inside the gut is another approach of assessing the influence of diet on improving gut health. Figure 7 shows the production of toxins inside the typical gut subjected to a different diet. Figure 7A shows the production of toxins inside the gut microbiome-harmful model, i.e., gut with harmful bacteria inside. On the other hand, Figure 7B shows that after adding beneficial bacteria into the microbial consortia, the level of toxins was significantly reduced in the case of the high-fiber diet. This suggests the need for both beneficial and high fiber diets to improve autism symptoms inside the gut.

### Microbiome and Diet Influence Intestinal and Metabolic Functions in Autism

**FIGURE 8:**
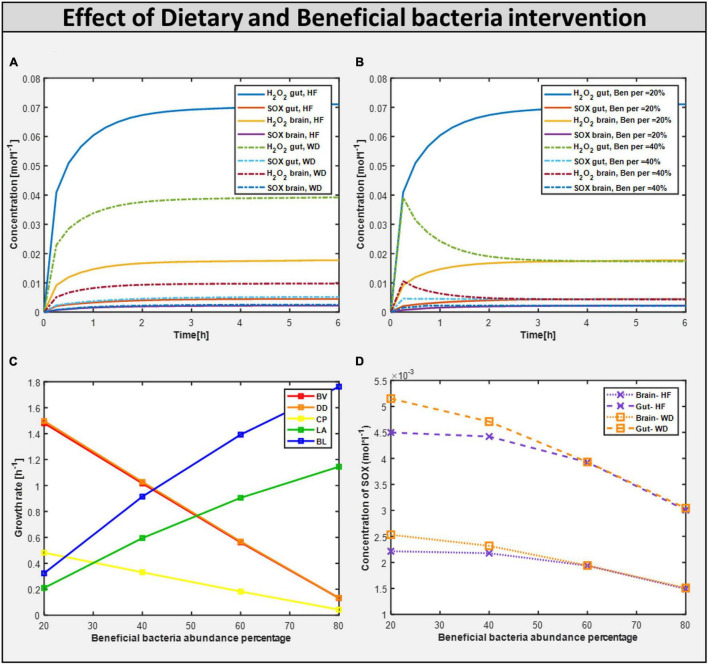
Effect of dietary and probiotic intervention. **(A)** Toxins profile in 20% abundance of beneficial bacteria in the community with change in diet from HF to WD. **(B)** Toxins profile with varying beneficial bacteria abundance in the community from 20% to 40% with fixed diet HF. **(C)** Growth rate of different bacterial biomass at differential abundance percentage. **(D)** Reducing the build-up of ROS toxin i.e. Superoxide (SOX) with the help of increasing beneficial percentage along with the correct diet.

Now, to better understand how the addition of beneficial bacteria to microbiome consortia improves autism symptoms in the gut, we explored another way of intervention: adding beneficial bacteria at various levels of intervention. To examine the effect of helpful bacteria, we introduced them to the purely autistic gut model, i.e., gut microbiome-bad bacteria, to create a model with varied levels of beneficial bacteria intervention, as shown above in model analysis, and found that the beneficial bacteria grew at a faster rate. Figure 8C depicts the growth rate of several bacteria in the gut model. Contrastingly, the addition of beneficial bacteria led to reduced flux in reactions linked to oxidative stress metabolism. Therefore, the concentration of ROS toxin is significantly reduced, as shown in Figure 7B.

### Changing Gut Microbial Composition With Beneficial Bacteria Improved Autistic Characteristics

By quantifying the level of toxins reaching the brain that are produced in the gut, the involvement of bacteria-derived toxins/metabolites in autism was identified. The toxins produced/consumed are allowed to reach a steady-state value before being fed to PBPK for whole-body toxin distribution in each time instant. The concentration profile of toxins in the gut and brain models fed with a high-fiber diet and 20% abundance of beneficial bacteria in the gut model are shown in Figures 8A,B, respectively. By increasing the fraction of beneficial bacteria in the gut, we observed a monotonic decrease in the growth rate of all harmful bacteria under both diets, as well as a monotonic decrease in the level of oxidative stress (Figure 8) in both the brain and the gut, which is equivalent to administering probiotics to an individual with autism.

### Neurotransmitters in the Integrated Brain Model

Studies and assessments on elevated levels of reactive oxygen species in neural tissues reveal that proteins involved in proper brain function are susceptible to oxidative changes, which alter their action. This is measured by determining the amount of GABA neurons in the brain that have been damaged by oxidative toxins.

**FIGURE 9:**
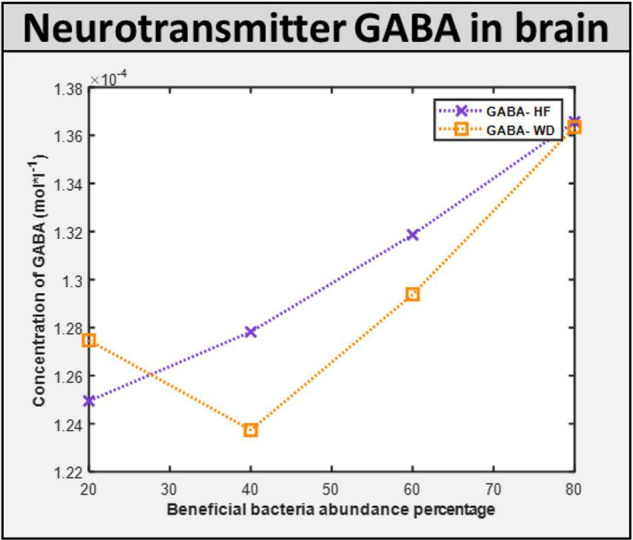
ROS induced level of neurotransmitter in the brain. GABA concentration in the brain with different diet HF and WD and increased level of probiotic intervention in the corresponding gut model.

Maintenance flux for each of the neuronal cell types in the brain model was obtained (Supplementary Files/Additional File 1/Table S16). Additionally, the model indicated that the flux through synthesis reactions of GABA, an inhibitory neurotransmitter, is increased upon intake of beneficial bacteria, as shown in Figure 9. This demonstrates that by increasing the level of probiotic intervention, the level of GABA required for normal brain activity may be retained, supporting studies on GABA dysfunction in autism (detailed in Supplementary Files/Additional File 1).

### Bacterial Secretion Products

The importance of core biochemical mechanisms driving autism must be understood, and metabolic biomarkers or bacterial secretion products in the gut can be used to assess them. The importance of an autistic-specific metabotype, i.e., secretion profile of a group of patients with autism group was realized *via* recent metabolomics studies (Yap et al., 2010; Ming et al., 2012) and other screening tests (Frye et al., 2013). We predicted a total of 230 possible bacterial secretion products (Supplementary Files/Table 3). The top 15 secretion products, i.e., the exchange reactions with highest maximum fluxes in the FVA are given in Supplementary Files/Additional File/ Table S17, ranked from high to low. The biomarker predictive capacity of the integrated model is moderate, as it captures some important bacterial secretion products implicated in autism, like propionic acid (MacFabe et al., 2007; MacFabe, 2012), lactate, and pyruvate, which contribute to conditions linked to autism, such as mitochondrial dysfunction (Rossignol and Frye, 2012) and oxidative stress (Rossignol and Frye, 2012), as well as ammonia (Ding et al., 2017). Although experimental validation of the remaining predicted bacterial secretion metabolites would be helpful, this is beyond the scope of this study.

We then analyzed the effect of the addition of probiotics on these potential biomarkers. The flux through harmful bacteria secretion is reduced as the fraction of beneficial bacteria is increased, as shown in Table 3 and Supplementary Files/Table 4. Typical examples include reduced flux through ammonia excretion. Interestingly, elevated levels of ammonia have been reported to be present in children with autism (Gorker and Tuzun, 2005; Ding et al., 2017).

**TABLE 3:** Summary of the effect of beneficial bacteria and diet on secretion products of the harmful bacteria.

Rank	(a) Harmful bacteria products	(b) Effect of probiotic addition	(c) Change	(d) Effect of diet change
1	Formate	Formate	Significant decrease	Hydrogen
2	Hydrogen	Ammonium	Decrease	Carbon dioxide
3	Acetate	Acetate	Decrease	Hydrogen ions
4	Ammonium	Hydrogen ions	Increase	Acetate
5	Guanine	Guanine	Decrease	Succinate
6	Carbon dioxide	Carbon dioxide	Increase	Pyruvate
7	Hydrogen ions	Hydrogen	Increase	Aminoacetaldehyde
8	Adenine	Xanthine	Increase	Lactate
9	Succinate	AMP	Output to input	Formate
10	Propionate	Glycerol 3-phosphate	Output to input	Malate
11	Lactate	Hydrogenphosphate	Output to input	Fumarate
12	Ethanol	Pyruvate	Increase	Ammonia
13	Aminoacetaldehyde	L-alanine	Decrease	Propionate
14	Pyruvate	Aminoacetaldehyde	Increase	2-Oxobutanoate
15	Malate	Adenine	Decrease	Ethanol

*Only top 15 bacterial secretion products are shown. (a) Harmful bacterial secretion products, (b) Harmful bacterial secretion products that are affected by the addition of beneficial bacteria, (c) change observed upon the addition of corresponding beneficial bacteria, (d) increased secretion products after changing the diet from Western to high-fiber.*

Furthermore, given these potential biomarkers, we analyzed their modulation *via* different diets. With the Western diet, the beneficial bacteria secreted 20 metabolites, 65% of which were foreign. With the high-fiber diet, beneficial bacteria secretion increased to 25 metabolites, 68% of which were foreign (Supplementary Files/Table 4). This is indicative of probiotics being relatively more effective in the high-fiber diet than the Western diet.

With the Western diet, the harmful bacteria secreted 45 metabolites, 76% of which were foreign to the gut lumen. The beneficial bacteria secreted 20 metabolites, 65% of which were foreign. With the high-fiber diet, the harmful bacteria secreted 48 metabolites, 79% of which were foreign to the gut lumen. The beneficial bacteria secreted 25 metabolites, 68% of which were foreign. These are indicative of probiotics being relatively more effective in the high-fiber diet than the Western diet. Since beneficial and harmful bacteria secrete common metabolites (Supplementary Files/Table 3), net bacterial secretion products in the gut microbiome model, with the Western diet, were found to be 45, 78% of which were foreign. With the high-fiber diet, net secretion was found to be 48 metabolites, 79% of which were foreign. These also show that bacterial secretion products themselves are dependent on the diet and not just their reaction fluxes. The top 15 bacterial secretion products for the harmful bacteria, i.e., exchange reactions with highest maximum fluxes in the FVA, are given in Supplementary Files/Additional File/Table S9, ranked from high to low. The entire list, along with their maximum secretion fluxes used to determine the ranking, is given in Supplementary Files/Table 5.

### Effect of Gut Microbiome and Diet on Gut Metabolism

The effect of addition of harmful gut bacteria on gut metabolism is determined by comparing the fluxes of sIEC reactions of the models gut microbiome–harmful and sIEC by FVA as before. Similarly, to determine the effect of addition of beneficial bacteria to this system, we compare the gut microbiome-harmful and gut microbiome models. We compare the gut microbiome using the Western and high-fiber diets to analyze the effect of diet. The reactions, ordered as per the extent to which they are affected, are given in Supplementary Files/Table 6.

The number of reactions affected in each of the comparisons is summarized in Supplementary Files/Additional File 1/Table S18.

### Effect of Gut Bacteria, Diet, and Reactive Species Metabolites on the Metabolism of Gut and Brain: Metabolic Pathway Analysis

**FIGURE 10:**
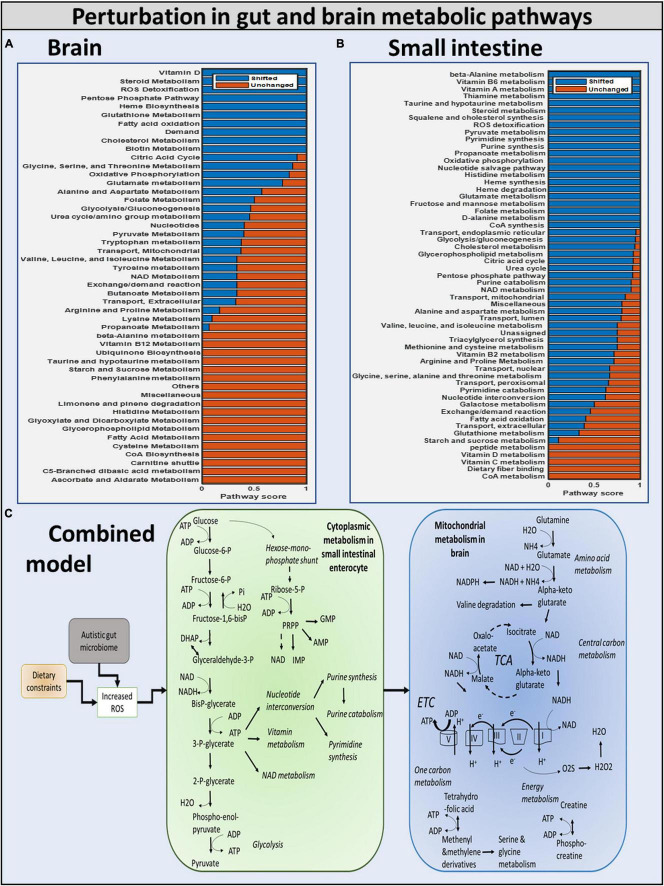
Summary of the modulation of metabolic pathways in autism. **(A)** Brain model: brain metabolic pathways perturbed due to dietary intervention. **(B)** Small intestinal model: SIEC metabolic pathways perturbed. **(C)** Combined model: overall shift in metabolism of brain and SIEC host.

We investigated if oxidative stress is reduced with the administration of probiotics and observed that steady-state SOX levels in the brain and gut are reduced with both diets, with the reduction in SOX larger with the high-fiber diet than with the Western diet, confirming our hypothesis that the Western diet is more harmful for autism pathogenesis, as shown in Figure 10 and Supplementary Files/Additional File/Table S6. The effect of varying percentages of beneficial bacteria on ROS levels is presented in Supplementary Files/Table 7. Consistent with the previously observed effect of the addition of probiotics, we observed that increasing the percentage of beneficial bacteria decreases the steady-state concentration of superoxide in the gut and brain. As indicated in Supplementary Files/Additional File 1/Table S18, several pathways affected by the addition of beneficial bacteria, i.e., pathways that are most affected by gut imbalance seen in autism, overlap with pathways affected by oxidative stress.

Purine catabolism and nucleotide interconversion are among the most affected pathways in autism pathogenesis (Supplementary Files/Additional File 1/Table S18). Consequently, several experimental studies have indicated the association of nucleotide metabolism with autism. Additionally, upon inclusion of beneficial bacteria to the purely autistic gut model, higher flux through glycolysis was observed, hinting toward the potential of this being one of the protective mechanisms against oxidative stress.

To determine the effect of oxidative stress on metabolic pathways affected in autism, we developed and compared the “toxic model” and the “non-toxic model” for both the gut and the brain, with the former allowing high exchanges of oxidative stress-related toxins across membranes because of increased permeability observed in autistic conditions (de Magistris et al., 2010; Fiorentino et al., 2016), and the latter corresponding to low toxin exchange. The pathways affected by oxidative stress are given in Supplementary Files/Table 7.

## Discussion

This is the first time that a hybrid modeling technique for ASD combining steady-state and dynamic modeling techniques has been presented. The integrated model closely resembles known metabolic abnormalities in gut and brain of people with autism. A novel dietary regimen for the treatment of autism symptoms is also recommended. The basic problem with merging constraint-based and PBPK models is that the former predicts reaction fluxes (rates), while the latter calculates reaction concentrations of the metabolites of interest. This was resolved by employing the static optimization strategy of dynamic Flux balance analysis dFBA (Mahadevan et al., 2002), which assumes that a reaction consumes the whole concentration of a metabolite in each time step; consequently, the maximum rate served as input reaction boundaries. Simulating multicellular systems is difficult for a variety of reasons. As a result, pareto-optimization was devised and used to find a solution with non-zero biomass fluxes for all cells (where possible), allowing the system to grow collectively. Important findings on the impact of diet and gut bacteria on autism include: (i) a high-fiber diet reduces the growth of harmful bacteria, lowering the amount of toxins produced in the gut; and (ii) probiotic intervention with beneficial gut bacteria lowers the level of toxins in the autistic gut and brain.

Metabolic interactions drive microbial communities. Modeling and understanding of microbial metabolism can disclose vital knowledge of how microorganisms form communities, interact, and can be employed in biotechnological processes as a result. Several methods for modeling microbial metabolism have been developed in recent decades, and some of them are being extended to represent microbial communities (Ibrahim et al., 2021). While there are alternative approaches to modeling microbial metabolism, the constraint-based modeling paradigm is, by far, the most used. Constraint-based modeling is a method for simulating any biological system, be it a single organism or a collection of species (Schellenberger et al., 2011).

We chose five and ten bacteria that have been linked to the autistic gut in previous studies, and a genome-scale metabolic reconstruction is also provided (Sandler et al., 2000; Finegold, 2008; Finegold et al., 2010; Frye and Rossignol, 2011; Ming et al., 2012; Frye et al., 2015; Ding et al., 2017; Shaaban et al., 2017; Vuong and Hsiao, 2017). To this end, we used the metabolic network of Desulfovibrio desulfuricans subsp. desulfuricans, Clostridium perfringens ATCC 13124, Lactobacillus acidophilus ATCC 4796, Bifidobacterium longum longum JDM301, Akkermansia muciniphila ATCC BAA 835, Clostridium difficile NAP07, Prevotella ruminicola 23, Ruminococcus torques ATCC 27756, and Shigella flexneri 2002017.

In systems biology, it is common to put together a consortium of disease-causing bacteria to better understand how they interact with one another and with the host. However, the growing demand for metabolic models of various bacteria necessitates the use of the methodology provided with a consortium of 776 gut microorganisms to make the forecast more realistic (Magnúsdóttir et al., 2016; Bauer and Thiele, 2018). However, those enormous-scale models with large-size models (millions of metabolic reactions from different microorganisms and hosts) will necessitate the most up-to-date big data approaches to handle the large amounts of data and extract insights from the results.

### Importance of the High-Fiber Diet in Reducing Autism Symptoms of Patients With Autism Spectrum Disorder Compared to the Western Diet

Because gut bacteria metabolize most of the fiber in the body, proliferation of all bacteria was shown to be generally higher with the high-fiber diet than with the Western diet (Table 2B; Slavin, 2013). As a result, the high-fiber diet encourages the growth of healthy gut bacteria. When compared to the Western diet, the high-fiber diet causes more bacterial secretion products to be produced (Table 3). As previously stated, gut bacteria are known to digest the majority of fiber in our diets, promoting bacterial development and resulting in more bacterial secretion products. We can deduce that the high-fiber diet is more effective than the Western diet in reducing autism symptoms.

### Significance of Beneficial Bacteria Intervention

Upon increasing the fraction of beneficial bacteria in the gut, which is equivalent to administering probiotics to an individual with autism, we observed a monotonic decrease in the growth rate of all harmful bacteria with both diets, and a monotonic reduction in the levels of SOX in both the brain and the gut (Figure 7). However, the production of H_2_O_2_ is reduced, where the combination of high-fiber diet and probiotic works significantly well in reducing the level of H_2_O_2_ until the percentage abundance of beneficial bacteria reaches 40%. Increasing further the abundance of beneficial bacteria increases the level of H_2_O_2_ produced, the reason being some of the metabolic pathways synthesizing SOX*to*H_2_O_2_ carry an increased flux in the model at a higher percentage of probiotic intervention, and one of the important enzymatic reactions synthesizing SOX is superoxide dismutase, which is also reported to be significantly lower in children with autism (Zoroglu et al., 2004). Superoxide dismutase is a potent protective enzyme that can selectively scavenge the radical ${\text{O}}_{2}^{-1}$ by catalyzing its dismutation to H_2_O_2_ :


(9) $$2\,H+\,2\,O_{2}\,\text{S}->{\text{O}}_{2}+H_{2}\,{\text{O}}_{2}$$

The flux through the superoxide dismutase in the host SIEC model increases with increase in probiotic composition, thereby increasing the level of H_2_O_2_ toxin in the host model. This is carefully analyzed and documented in the five-microbe_community/Supplementary Files/Table 7.xlsx. Furthermore, the flux through other pathways associated with ASD condition, such as thiobarbituric acid-reactive substances (TBARS), superoxide dismutase (SOD), and catalase (CAT) xanthine oxidase (XO), adenosine deaminase (ADA) affected in the model with the change in the probiotics composition in the community model can be followed in Additional_file_8.xlsx.

### Brain Adenosine Triphosphate and Neurotransmitter Levels

Altered neurotransmissions in GABAergic neurons, including loss of GABA interneurons, have been identified in a subset of autism cases (Coghlan et al., 2012; Chen et al., 2015; Vuong and Hsiao, 2017). Hence, we next simulated the effect of probiotics on brain metabolism. Maintenance flux for each of the neuronal cell types in the brain model was obtained (Supplementary Files/Additional File/Table S16). Additionally, the model indicated that the flux through the synthesis reactions of GABA, an inhibitory neurotransmitter, was increased upon intake of beneficial bacteria (Supplementary Files/Table 5). Hence, we propose that probiotics may improve autism, majorly *via* increasing the availability of GABA in the brain.

They investigated the evidence related to the hypothesis that ASD is characterized by dysfunction in the GABA signaling mechanism. Furthermore, they attempted to explain how the abnormality theoretically manifests in autism symptoms by performing genetic and *in vivo* studies. The results produced by the integrated model also show marked deviation in GABA levels in the brain of individuals with “purely autistic” gut when compared with the “corrected” gut, thus concurring with the study of Coghlan (Coghlan et al., 2012).

### Modeling Oxidative Stress Is Important for Understanding Fundamental Metabolic Mechanisms That Cause Autism

Typically, the production of ROS, H_2_O_2_, and SOX(Bjørklund et al., 2020) is associated with autism (MacFabe, 2012). To determine the effect of oxidative stress on metabolic pathways affected in autism, we developed and compared the “toxic” model and the “non-toxic” model for both the gut and the brain, with the former allowing for high exchanges of oxidative stress-related toxins across membranes because of increased permeability observed under autistic conditions (Kondoh et al., 2007; Ming et al., 2012; Downs et al., 2014), and the latter corresponding to low toxin exchange.

The toxin concentration gradually increased during the simulation until it reached saturation (Figure 8). This occurred because the simulation starts with initial conditions chosen for an ideal (or desired) case of very low toxin concentrations in the body, which would then naturally increase with the activation of metabolic pathways, producing ROS in the gut and the brain, reaching saturation levels where the net intake and production of ROS from the body balanced the net consumption and excretion of ROS from the body. The H_2_O_2_ toxin profile in organs is reduced to 40% beneficial percentage in the presence of a high fiber diet. The H_2_O_2_ toxin increases steadily with the Western diet. This highlights that high-fiber diet and optimum number of probiotics are important to reduce the toxins produced in autism.

**FIGURE 11:**
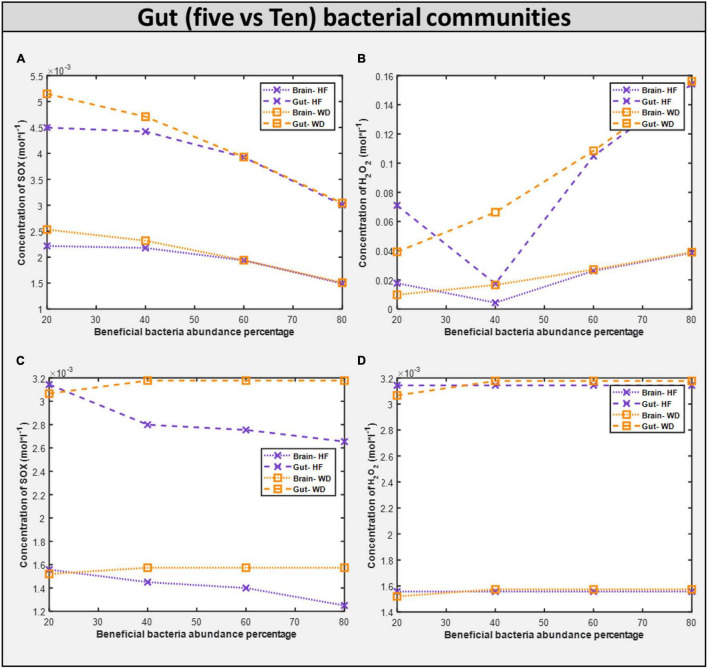
Comparison of toxins (H_2_O_2_ and O_2_S) in five vs ten bacterial gut representative model and toxins in corresponding brain model. **(A)** SOX concentration generated in five microbial community. **(B)** H_2_O_2_ concentration generated in five microbial community. **(C)** SOX concentration generated in ten microbial community. **(D)** H_2_O_2_ concentration generated in five microbial community.

First, upon increasing the fraction of beneficial bacteria in the gut, which is equivalent to administering probiotics to an individual with autism, we observed a monotonic decrease in the growth rate of all harmful bacteria with both diets, and a monotonic reduction in the levels of oxidative stress in both the brain and the gut (Figure 11). The correlation between these two phenomena is further highlighted by the presence of glutamate metabolism and ROS detoxification among the most affected pathways in the brain (McGinnis, 2004; Table 4).

**TABLE 4:** Top 10 gut metabolism pathways affected by harmful bacteria subsequent addition of beneficial bacteria, and change in diet, and effect of oxidative stress on the gut and brain.

S. No.	Pathways affected by addition of beneficial bacteria to “purely” autistic gut	Pathways affected by change in diet	Pathways affected in the gut	Pathways affected in the brain
1	Purine catabolism	Purine catabolism	Purine catabolism	ROS detoxification
2	Nucleotide interconversion	Glycolysis/gluconeogenesis	Citric acid cycle	Oxidative phosphorylation
3	Glycolysis/gluconeogenesis	Fructose and mannose metabolism	Alanine and aspartate metabolism	Pyruvate metabolism
4	Citric acid cycle	Citric acid cycle	Purine synthesis	Glutamate metabolism
5	Valine, leucine, and isoleucine metabolism	Pyruvate metabolism	Vitamin B6 metabolism	NAD metabolism
6	Propanoate metabolism	Nucleotide interconversion	NAD metabolism	Citric acid cycle
7	Pyruvate metabolism	NAD metabolism	Pyrimidine catabolism	Urea cycle/amino group metabolism
8	Vitamin B6 metabolism	Purine synthesis	Glycolysis/gluconeogenesis	Alanine and aspartate metabolism
9	NAD metabolism	Vitamin B6 metabolism	Arginine and proline metabolism	Valine, leucine, and isoleucine metabolism
10	Purine synthesis	Pyrimidine catabolism	Nucleotide interconversion	Folate metabolism

### Autistic Biomarkers Are Revealed by Metabolic Perturbations in Gut and Brain Models

Diet and gut microorganisms have been found to have a significant impact on host metabolism, primarily through central carbon, nucleotide, and vitamin metabolism in the gut, and mitochondrial energy, amino acid, and metabolism in the brain (Figure 10). While cytoplasmic pathways were discovered to be severely impacted in the small intestine enterocyte model, mitochondrial metabolism was found to be disrupted in the brain. Because ATP is involved in glycolysis, nucleotide, and vitamin metabolism, these pathways were discovered to be not only interconnected, but also to be the primary players in mediating small intestinal metabolism in the autistic instance. In the brain, glutamate and glutamine metabolism was discovered to be interconnected with downstream processes, such as energy generation *via* the TCA, ETC, and phosphocreatine pathways, as well as one-carbon metabolism. As a result, our model revealed a greater flux through glutamate metabolic reactions in the brain. Autism has been linked to high glutamate levels and a malfunctioning mitochondrial glutamate transporter (Yap et al., 2010; Ding et al., 2017). Figure 10 shows a summary of the numerous metabolic impacts. Finally, the model was utilized to demonstrate that by including probiotics in one’s diet, altered intestine and brain metabolism can be managed to alleviate autism symptoms. Reduced ROS levels in the gut and increased GABA levels in the brain led to this conclusion.

Studies have associated abnormal amino acid, fatty acid, energy, and reactive species metabolism (Frye et al., 2015; Cheng et al., 2017) with autism. Herein, we report that the purine catabolism and nucleotide interconversion pathways are among the most affected pathways in autism pathogenesis when simulated with the Western diet (Supplementary Files/Additional File/Table S10). Consequently, elevated purine catabolism *via* xanthine oxidase (GeneID: 7498, *XDH*, E.C. 1.17.3.2.) was reported on patients with autism (Zoroglu et al., 2004). Further, increased uric acid levels in children with autism, and anti-purinergic therapy has been shown to reduce autistic features in mice (Naviaux et al., 2014).

Pyruvate and lactate, both known biomarkers of mitochondrial dysfunction, are present at abnormally high levels in individuals with autism (Rossignol and Frye, 2012). The integrated model was also in consonance with this evidence from the literature, wherein metabolic pathways of pyruvate and lactate metabolism were among the most affected in models of autism (Supplementary Files/Additional File/Tables S14, S15). An interesting observation shown in Supplementary Files/Additional File/Table S17 is that among the top three secretion products, the Bacteroides vulgatus model contributed the maximum amount to the total formate and acetate generated in the autistic gut (Supplementary Files/Additional File/Table S17). This makes sense, since Bacteroides vulgatus decomposes complex sugars and produce short-chain fatty acids (Wexler, 2007). On the other hand, the Clostridium perfringens model generated maximum amount of H_2_. Thus, *Bacteroides vulgatus* and *Clostridium perfringens* can be the most severe drivers of autism among all the implicated harmful bacteria, and the significant reduction in the levels of formate in the gut upon addition of beneficial bacteria (Supplementary Files/Additional File/Table S17) further strengthen this theory. In fact, we propose that development of novel drugs and dietary formulations focusing on reducing the quantities of these two bacteria can show huge improvement in autism symptoms. Simultaneously, we propose novel biomarkers for autism (Supplementary Files/Additional File/Table S17), warranting clinical investigations.

### Benefit of Increasing Gut Microbiota Community to Ten Bacteria

The large-scale dynamics of the microbiome can be used to better understand population ecology and microbial community characteristics (Chen et al., 2015). Increasing the size of model allows us to better understand the behavior of our new framework and demonstrate its durability in the face of a more complicated and extended flux-based model. We used two different-sized microbial community models. An extended model was utilized to see if the conclusions from the five-microbe community model on metabolic pathways, oxidative stress, secretory profile, and treatment alternatives are still valid.

The community is carefully observed, and it was found that the microbe *Desulfovibrio desulfuricans* changed its phenotype behavior because of changes in its interacting partners. Interestingly, abundance of *Desulfovibrio desulfuricans* in the stool of patients with autism has been reported, and the change in abundance is positive (Finegold, 2011), i.e., patients with autism have an increased abundance of *Desulfovibrio desulfuricans* in the gut, which means that it should not be taken as beneficial bacteria but instead as harmful bacteria. Therefore, when increasing the beneficial bacteria, we are also increasing *Desulfovibrio* abundance. Hence, the effect from the beneficial bacteria and *Desulfovibrio* nullifies the total beneficial effect. This demonstrates the resilience of the model and importance of selecting the proper probiotic.

Dietary intervention in autism is crucial for both the GI and autistic systems to improve (Kang et al., 2017). Steady-state SOX concentrations were observed to be lower with the high-fiber diet as than with the Western diet according to the five-microbe community model. In the presence of a high fiber diet, the H_2_O_2_ toxin profile in organs is lowered to a 40 percent favorable percentage. Like in the five-microbe community model, steady-state SOX concentrations were found to be lower for the high-fiber diet than for the Western diet. This emphasizes the importance of a high-fiber diet, as well as proper dosage and selection of probiotics, in reducing the toxins produced in autism.

The growth rate of bacteria in the ten gut-microbial community is higher with the high-fiber diet than with the Western diet (Supplementary Files/Additional File/Table S4), confirming our findings from five-microbe community studies (Slavin, 2013). Upon increasing the fraction of beneficial bacteria in the gut, which is equivalent to administering probiotics to an individual with autism, we observed a monotonic decrease in the growth rate of all harmful bacteria with both diets like in the five-microbe community, and a monotonic increase in the levels of *SOX* in both the brain and the gut unlike in the case of the five-microbe community. Furthermore, the flux through which other pathways associated with the ASD condition, such as thiobarbituric acid-reactive substances (TBARS), superoxide dismutase (SOD), catalase (CAT), xanthine oxidase (XO), and adenosine deaminase (ADA), affected the model with change in probiotic composition in the community model can be found in Supplementary Files/Table 7. In addition to the purine catabolism and nucleotide interconversion pathways that were found to be affected in the five-microbe community model, we also found glutathione metabolism to be among the most affected pathways in autism pathogenesis (Kurochkin et al., 2019; Supplementary Files/Additional File/Table S8).

## Conclusion

The novel *in silico* approach used in this study demonstrates how toxins produced by gut dysbiosis alter the normal function of the brain in ASD. Integrating the steady-state and dynamic techniques (i.e., COBRA and PBPK, respectively) has enabled the development of the novel hybrid model, a whole-body transport system with tissue-specific metabolic details. Tissue-specific models revealed metabolic disruption in both the gut and the brain. Most importantly, we showed the integral connection between diet and gut microbes that majorly influences host metabolism, typically *via* central carbon, nucleotide, and vitamin metabolism in the gut, and mitochondrial energy, amino acid, and metabolism in the brain. This approach helped in gaining better insights into the gut-brain axis in ASD. By leveraging the varying compositions of gut bacteria models, i.e., five-bacteria gut model and ten-bacteria gut model, we found that *Bacteroides vulgatus* and *Clostridium perfringens* contribute maximally to total formate and acetate generated in the autistic gut. Hence, these were identified to be the severe drivers of autism among all the implicated harmful bacteria. In response to dietary intervention, a high-fiber diet combined with beneficial bacteria, i.e., *Lactobacillus acidophilus* and *Bifidobacterium longum*, is proposed to help alleviate autism symptoms. The unique paradigm we provide here can, thus, be used for metabolic and metabolomic investigations on diseases characterized by disturbed microbial makeup in the gut.

## Data Availability Statement

The original contributions presented in the study are included in the article/Supplementary Material, further inquiries can be directed to the corresponding author.

## Author Contributions

FM, MP, and SSh built the models, analyzed the data, and interpreted the results. FM and MP developed the algorithms and analysis tools. RR monitored the PBPK modeling. SS was responsible for the COBRA modeling parts, conceived and designed the study, and planned the experiments. All authors wrote the manuscript.

## Conflict of Interest

The authors declare that the research was conducted in the absence of any commercial or financial relationships that could be construed as a potential conflict of interest.

## Publisher’s Note

All claims expressed in this article are solely those of the authors and do not necessarily represent those of their affiliated organizations, or those of the publisher, the editors and the reviewers. Any product that may be evaluated in this article, or claim that may be made by its manufacturer, is not guaranteed or endorsed by the publisher.

## Abbreviations

COBRA: constraint-based regression and analysis
PBPK: physiologically based pharmacokinetic model
ASD: autism spectrum disorder
GABA: gamma-aminobutyric acid
FVA: flux variability analysis
SIECs: small intestinal enterocyte cells
ROS: reactive oxygen species
*SOX*: superoxide
*H*_2_ *O*_2_: Hydrogen peroxide
AGORA: assembly of the gut organism through reconstruction and analysis
QSPR: quantitative structure-property relationship
ATP: adenosine triphosphate
BV: Bacteroides vulgatus ATCC8482
DD: Desulfovibrio desulfuricans subsp. desulfuricans DSM642
CL: Clostridium perfringens ATCC13124
LA: Lactobacillus acidophilus ATCC4796
BL: Bifidobacterium longum longum JDM301
AKK: Akkermansia muciniphila ATCCBAA835
CL: Clostridium difficile NAP07
PR: Prevotella ruminicola 23
RT: Ruminococcus torques ATCC27756
SH: Shigella flexneri 2002017.
