# Supplementary material for: A Computational Framework for Studying Gut-Brain Axis in Autism Spectrum Disorder
Source: Front Physiol. 2022 Mar 7;13:760753. doi: 10.3389/fphys.2022.760753 (PMC8940246; doi:10.3389/fphys.2022.760753)
Supplement: Supplementary file 1 [file Data_Sheet_1.docx]

**Additional file 1**

**A computational framework for studying metabolic abnormalities and the role of diet and gut microbiota in autism spectrum disorder**

Faiz Khan Mohammad^1^, Meghana Venkata Palukuri^2^, Shruti Shivakumar^1,2^, Swagatika Sahoo^1,2^, Raghunathan Rengaswamy^1,2*^

**Autism - gut relationship**

A recent pyrosequencing study (Finegold et al., 2010) has shown that the genera Bacteroides and Desulfovibrio are present in significantly higher amount in the gut of autistic individuals than controls while Bifidobacterium and Lactobacillus, known to be the 'protective bacteria' due to their anti-oxidative effects and their usage as probiotics to alleviate the symptoms of gastro-intestinal diseases are present in reduced quantities. In fact, administering fructo-oligosaccharides as a prebiotic has been proposed to increase the concentrations of Bifidobacterium (Weston et al., 2015).

Increasing evidence has shown that oxidative stress plays a major role in the development of autism (Chauhan and Chauhan 2006). It results in altered sulphuration pathways (James et al., 2004), which further increases the concentrations of Desulfo in the gut microbiome due to its sulphur metabolism capability (Heberling et al., 2013). Such an increase results in a reduction in the levels of the anti-oxidant bacteria, Bifidobacterium, which compounds the effects of oxidative stress (Newton et al., 1998). Oxidative stress also causes lower levels of the glutathione, a known anti-oxidant, in autistic individuals (James et al., 2004).

**Leaky Gut hypothesis**

The Leaky gut hypothesis refers to the inability of the gut epithelial cells to discern between molecules passing from the gut to the bloodstream and vice versa, caused due to the widening of the tight junctions in the gut wall. One explanation for such intestinal inflammation is the increased levels of toxins like propionic acid (released by *Bacteroides*) and lipo-polysaccharide (released by *Desulfovibrio*), which trigger the production of pro-inflammatory cytokines. This increases gut permeability allowing the toxins into the bloodstream, which can further breach the blood-brain barrier and cause autistic characteristics (de Theije et al., 2011). The experiments by MacFabe et al. (MACFABE et al., 2007) (MacFabe, 2012) reaffirmed this hypothesis by observing ASD-like symptoms after injecting propionic acid in the brains of rats. The computational simulation by Downs et al. (Downs et al., 2014) showed increased concentrations of propionic acid and cytokines in the brain due to leaky gut syndrome.

Recent studies have demonstrated that Reactive Oxygen Species (ROS) are also implicated in intestinal inflammation observed in inflammatory bowel diseases like Crohns disease and peptic ulcers (Balmus et al., 2016; Bhattacharyya et al., 2014).

**Constraint-based model construction**

Each of the bacteria models was corrected for errors in the naming of exchange reactions (inconsistencies in metabolite names and their corresponding exchanges). Additional exchange reactions were added for extracellular dead-end metabolites to ensure their corresponding reactions could carry flux. For easy access to the formulas for each of the reactions, a rxnFormulas field was created. The models were tested for growth using Flux Balance Analysis (FBA) (Orth et al., 2010) , by maximizing biomass reaction flux under the western diet and high-fiber diet and growth rates consistent with those of the AGORA analysis were obtained in both aerobic and anaerobic conditions. Further, to be consistent with steady state conditions, a leak test was performed to ensure that there was no net production of any metabolite for zero input fluxes.

SIEC model post-processing was done in a manner similar to the bacterial models - exchange reactions were corrected, rxnFormulas field was added and a test for growth (biomass production) and model leakage test was done.

The individual neuronal models were corrected so that the naming of metabolites and exchange reactions was consistent with those of the gut microbiome models. Further, an additional field rxnFormulas was added and the model was checked for any leaks.

**COBRA methods**

We present here additional COBRA functions developed for model quality checks and ease of usage.

*FVA_analysis* – Comparison of 2 models using FVA as described in the previous section

*write_FVA_res* – Writes the ordered reaction fluxes to a spreadsheet

*pareto_optim* – Finds the pareto optimal solution as explained in the previous section.

*correct_exchanges* – Renames exchanges that are inconsistent with metabolite names.

*add_dead_end_met_exc* – Adds exchange reactions for extracellular dead end metabolites

*model.rxnFormulas* – New field introduced in the COBRA model structure containing the formulas of each reaction for ease of analysis

*write_model* – Writes the reactions, metabolites and other corresponding important fields of a model to a spreadsheet

*LeakTest* – Check for any net production of any metabolite under no input conditions, to be consistent with steady state conditions

*constr_model* – constrain a model according to diet and intestinal conditions

*get_growth* – returns individual, sum and pareto optimal growth of a system with multiple components

*addRatioReactionMulti* – For setting inequality constraints with more than two reactions involved.

*rename_model* – For renaming model reaction and metabolite names by replacing/appending additional information, example – GABA instead of Neuron.

The functions addMetabolite, addReaction, addRatioReaction fluxVariability, mergeTwoModels, and removeMetabolites from COBRA toolbox (Schellenberger et al., 2011) have been modified for better usage in the present analysis.

**PBPK model equations and parameters**

The final set of equations to model the distribution of SOX is shown in the following equations.

Table S1:

|  |  |  |
| --- | --- | --- |
| Brain | $\frac{\mathbf{f}\mathbf{V}_{\mathbf{B}}}{\mathbf{1+f}}\frac{\mathbf{d}\mathbf{C}_{\mathbf{B}}}{\mathbf{dt}}\mathbf{=}\mathbf{Q}_{\mathbf{B}}\mathbf{C}_{\mathbf{ART}}\mathbf{-}\mathbf{Q}_{\mathbf{B}}\mathbf{C}_{\mathbf{B}}\mathbf{+}\frac{\mathbf{P}_{\mathbf{B}}\mathbf{S}_{\mathbf{B}}\mathbf{C}_{\mathbf{EVB}}}{\mathbf{K}_{\mathbf{B}}}\mathbf{-}\mathbf{P}_{\mathbf{B}}\mathbf{S}_{\mathbf{B}}\mathbf{C}_{\mathbf{B}}$  $\frac{\mathbf{V}_{\mathbf{B}}}{\mathbf{1+f}}\frac{\mathbf{d}\mathbf{C}_{\mathbf{EVB}}}{\mathbf{dt}}\mathbf{=}\mathbf{P}_{\mathbf{B}}\mathbf{S}_{\mathbf{B}}\mathbf{C}_{\mathbf{B}}\mathbf{-}\frac{\mathbf{P}_{\mathbf{B}}\mathbf{S}_{\mathbf{B}}\mathbf{C}_{\mathbf{EVB}}}{\mathbf{K}_{\mathbf{B}}}$ | 1a |
| Heart | $\frac{fV_{H}}{1+f}\frac{dC_{H}}{\mathrm{dt}}=Q_{H}C_{\mathrm{ART}}-Q_{H}C_{H}+ \frac{P_{H}S_{H}C_{\mathrm{EVH}}}{K_{H}}-P_{H}S_{H}C_{H}$  $\frac{V_{H}}{1+f}\frac{dC_{\mathrm{EVH}}}{\mathrm{dt}}=- \frac{P_{H}S_{H}C_{\mathrm{EVH}}}{K_{H}}+P_{H}S_{H}C_{H}$ | 1b |
| Adipose | $\frac{fV_{A}}{1+f}\frac{dC_{A}}{\mathrm{dt}}=Q_{A}C_{\mathrm{ART}}-Q_{A}C_{A}+ \frac{P_{A}S_{A}C_{\mathrm{EVA}}}{K_{A}}-P_{A}S_{A}C_{A}$  $\frac{V_{A}}{1+f}\frac{dC_{\mathrm{EVA}}}{\mathrm{dt}}=- \frac{P_{A}S_{A}C_{\mathrm{EVA}}}{K_{A}}+P_{A}S_{A}C_{A}$ | 1c |
| Liver | $\frac{fV_{L}}{1+f}\frac{dC_{L}}{\mathrm{dt}}=Q_{L}C_{\mathrm{ART}}-Q_{L}C_{L}+ \frac{P_{L}S_{L}C_{\mathrm{EVL}}}{K_{L}}-P_{L}S_{L}C_{L}$  $\frac{V_{L}}{1+f}\frac{dC_{\mathrm{EVL}}}{\mathrm{dt}}=- \frac{P_{L}S_{L}C_{\mathrm{EVL}}}{K_{L}}+P_{L}S_{L}C_{L}$ | 1d (metabolism rate of ROS) |
| Gut | $\frac{fV_{G}}{1+f}\frac{dC_{G}}{\mathrm{dt}}=Q_{G}C_{\mathrm{ART}}-Q_{G}C_{G}+ \frac{P_{G}S_{G}C_{\mathrm{EVG}}}{K_{G}}-P_{G}S_{G}C_{G}$  $\frac{V_{G}}{1+f}\frac{dC_{\mathrm{EVG}}}{\mathrm{dt}}=- \frac{P_{G}S_{G}C_{\mathrm{EVG}}}{K_{G}}+P_{G}S_{G}C_{G}$ | 1e (generation rate of ROS) |
| Kidney | $\frac{fV_{K}}{1+f}\frac{dC_{K}}{\mathrm{dt}}=Q_{K}C_{\mathrm{ART}}-Q_{K}C_{K}+ \frac{P_{K}S_{K}C_{\mathrm{EVK}}}{K_{K}}-P_{K}S_{K}C_{K}$  $\frac{V_{K}}{1+f}\frac{dC_{\mathrm{EVK}}}{\mathrm{dt}}=- \frac{P_{K}S_{K}C_{\mathrm{EVK}}}{K_{K}}+P_{K}S_{K}C_{K}$ | 1f |
| Circulation | $C_{\mathrm{VEN}}= \frac{Q_{B}C_{B}+Q_{A}C_{A}+Q_{L}C_{L}+Q_{G}C_{G}+Q_{K}C_{K}}{Q_{H}}$ | 1g |

The symbols used in the equations above are explained in Supplementary Table S2.

Note that in the above model, we have not included any compound generation, consumption (metabolism) or removal (excretion) terms. The rates of the aforementioned terms need to be simply added or subtracted to the total balance depending on the organ.

**Physiological parameters in PBPK model:**

Physiological parameters include,

Blood flow rate through different tissue types QT,

The volume of each compartment VV and VEV

Tissue fractional blood volume f, and

Cross-sectional surface area of each compartment S.

Values for all of these four parameters for each of the tissue types was obtained from literature, except for f and S of certain tissue types, as shown in Table S2. Tissue fractional blood volume for these tissues was approximated to that of the brain, and the surface area of these tissue types was approximately calculated by assuming they were spherical.

Estimation of physicochemical parameters in PBPK model:

The physicochemical parameters refer mainly to (i) the tissue-plasma partition coefficients of each compartment for each specific molecule, (ii) the renal clearance in the kidney as well as (iii) permeability of the epithelial tissue of each organ.

**Partition coefficient of tissue type,** $\mathbf{K}_{\mathbf{T}}$ :

The tissue-plasma partition coefficient is the relative equilibrium distribution of the given compound between the two phases. Thus,

$$\begin{aligned} K_{T}= \frac{C_{TEV}}{C_{TV}}\#\left( 2 \right) \end{aligned}$$

Partition coefficients are generally determined using *in vitro* methods, such as vial equilibration, equilibrium dialysis and ultrafiltration, and using *in vivo* methods (Lam et al., 1982). Since, experimental methods can lead to fallacious *K_T_* values if steady state is not achieved at the time of measurement, and can also be expensive and time-consuming, researchers are increasingly using *in silico* methods to compute *K_T_*. These algorithms use the chemical properties of the compound and the physiological and composition characteristics of the tissues to predict *K_T_*. The algorithms are mainly of two types - tissue composition based (TCB) algorithms (Rodgers et al., 2005) (Rodgers and Rowland, 2006) and correlation-based algorithms (Yun and Edginton, 2013) (Jansson et al., 2008). TCB algorithms are mechanistic in nature i.e. they rely on modeling the tissue composition and the complex interactions between the compound and the tissue, rather than any experimentally obtained *in vivo* data. Studies by Graham et. al (Graham et al., 2011) and Jones et. al (Jones et al., 2011) suggested that correlation based algorithms, especially the Janssons model (Jansson et al., 2008) have higher accuracy in *K_T_* prediction than the best TCB model, which was developed by Rodgers et. al. (Rodgers et al., 2005).

**Quantitative Structure-Property Relationship (QSPR):**

Inspired by the correlation-based approach, Quantitative Structure-Property Relationship (QSPR) was used to estimate KT. QSPR models assume a strong correlation between the structure of a compound and its physical and chemical properties like melting point, boiling point, and in our case, tissue-plasma partition coefficient. The fundamental assumption behind QSPR is that molecules with similar structure have similar observable properties, while molecules with different structures have different observable properties. QSPR models are built with predictors consisting of theoretical molecular descriptors and fragment descriptors of the compound, which are derived solely from the molecular structure of the compound and not from experimental data, and the output variable being the physicochemical property of interest. Thus,

Partition coefficient = f(molecular descriptors) + error

Several authors have used QSPR as a means to predict tissue-plasma partition coefficient (Liu et al., 2005; Katritzky et al., 2005), and similar steps were followed to estimate *K_T_*.

**Data collection and preparation - SMILES file generation**:

We manually curated a training dataset from Yun-Edington et. al. (Lam et al., 1982), Rodger et. al. (Coghlan et al., 2012), Rodgers et. al. (Orth et al., 2010), Jansson et. al. (Jansson et al., 2008) and Liu et. al. (Liu et al., 2005). The dataset varies in size according to the tissue type of interest (i.e., brain, heart, adipose, liver, gut, and kidney), the largest consisting of 140 compounds (Table S2). We then obtained the canonical SMILES encoding for these chemicals from PubChem (Varma et al., 2009). SMILES, or Simplified Molecular Input Line Entry Specification, is a linear text format which can describe the connectivity and chirality of a molecule. It is unique string that can be used as a universal identifier for a specific chemical structure.

**Data collection and preparation - descriptor generation :**

PaDEL-descriptor (Yap, 2010), a free and open source software was used to calculate 1444 1D and 2D descriptors. Five additional descriptors fraction of neutral lipids, neutral phospholipids, and water in the tissue of interest and also the tissue-plasma:albumin ratio and tissue-plasma:lipoprotein ratio were also added to ascertain their effect on partition coefficient, as was shown in tissue-composition based mechanistic models.

**Model generation - Regularized linear model:**

A sparse linear model was constructed using Lasso (Tibshirani, 1996) to shrink descriptors affecting the *K_T_*. It was observed that Lasso zeroes the coefficients of all the tissue-specific descriptors, i.e., the tissue compositions and the tissue-plasma albumin and lipoprotein ratios, which is justified since tissue-specific descriptors have the same value for all molecules and therefore cannot possible contribute to the determination of *K_T_*.

**Model prediction :**

Table S4 tabulates the partition coefficient generated by the model for each of the tissue types for the toxins of interest. The values of partition coefficient computed for propionic acid can be used in further investigations in the relationship between gut bacterial toxins and autism.

**Renal clearance, PT :** To compute renal clearance for SOX, a method similar to that used in computing partition coefficient was used, with a training dataset of renal clearance published by Varma et. al was used (Varma et al., 2009). The canonical SMILES encoding for these molecules was obtained from PubChem and PaDEL-descriptor was used to obtain descriptors. A regression tree was used to predict the renal clearance for propionic acid, hydrogen peroxide and SOX. Table S4 tabulates the values of renal clearance generated by the model.

**Permeability of tissue type, PT :** According to Overtons rule,

$$\begin{aligned} P_{T}= \frac{K_{T}D}{\Delta x}\#\left( 3 \right) \end{aligned}$$

where *D* is the diffusion coefficient for the diffusion of the molecule across the cell lipid wall, and ∆*x* is the thickness of the cell membrane. Lipid molecules diffuse freely through lipid bilayers, with a diffusivity of 10^−10^*dm*^2^*/sec* [20] and thickness of the cell membrane is 8 × 10^−8^ dm [21]. Thus, permeability of all tissue types, except the brain and gut due the leaky-gut hypothesis, was calculated using *K_T_* estimated in Table S4. To account for the increased permeability of the epithelial wall of the gut and brain, a methodology similar to Downs et. al (Downs, R., Perna, J., Vitelli, A., Cook, D., and Dhurjati, P. 2014) was adopted, and the apparent permeability was used for both the gut lumen and brain.

**Biochemical parameters in PBPK model:**

This includes the metabolism, i.e., the rate at which the chemical is consumed by macromolecular binding, generation and excretion rates in the PBPK model. Metabolism was assumed to be a saturable processes as shown in Equation 1.

$$\begin{aligned} \frac{dA_{met}}{dt}= \frac{V_{\max C_{T}}}{K_{m}+C_{T}}\#\left( 4 \right) \end{aligned}$$

where, *V_max_* is the maximum enzymatic reaction rate (mol/sec), *K_m_* is the Michaelis constant for enzymatic reactions (mol/l), *A_met_* is the moles of the chemical metabolized in the liver. The values of the kinetic parameters, *V_max_* and *K_m_* were obtained from literature (Karadag and Bilgin 2010) for *H*_2_*O*_2_ and SOX as shown in Table S2.

Note: Estimates of the various physicochemical, biochemical and physiological parameters in the PBPK model were obtained for rats due to the non-availability of a large number of parameter values for humans.

Table S2: Details of PBPK model. (a) Table of steps involved in PBPK modeling. (b) Symbols and subscripts used in the mathematical representation of the PBPK model. (c) Characteristic of each compartment in PBPK model. (d) Physiological parameters used in the PBPK model. (e) Train set matrix size for each tissue type - rows denote the compound and columns denote the descriptors. (f) Values of kinetic parameters used in the metabolism terms.

(a)

| 1 | Model representation: | Involves deciding the compartments and the metabolic pathways relevant to the compounds of interest. Also includes formulation of the mathematical equations governing each compartment and their mutual interaction |
| --- | --- | --- |
| 2 | Model parameterization: | Involves obtaining estimates of physicochemical, physiological and biochemical parameters used in the above model equations |
| 3 | Model simulation: | Solving the algebraic and differential equations formulated above to determine compound uptake in each compartment |
| 4 | Model evaluation & validation: | Comparing model predictions with experimental data and evaluating model sensitivity, uncertainty and variability |

(b)

| Subscripts | Description | Symbols | Description |
| --- | --- | --- | --- |
| V | Vascular part of the organ | C | Concentration |
| EV | Extravascular part of the organ | V | Volume |
| B | Brain | Q | Blood flow rate |
| H | Heart | K | Partition coefficient for ROS |
| A | Adipose | P | Permeability of tissue |
| L | Liver | S | Surface area of tissue |
| G | Gut | *CL_R_* | Renal clearance |
| K | Kidney |  |  |
| ART | Arterial blood |  |  |
| VEN | Venal blood |  |  |

(c)

| Physicochemical characteristics of tissue | Refers primarily to the water, lipid and phospholipid composition of each tissue. This would account for the solubility of the compound in the tissue |
| --- | --- |
| Biochemical characteristics of tissue | Refers to binding and metabolizing capability of tissue |
| Physiological characteristics of tissue | Refers to blood flow rate, volume, surface area, and other physiological properties of the tissue |

(d)

| Parameter type | Parameter | Value (*Lmin*^−1^) | Reference |
| --- | --- | --- | --- |
| Flow rate (Q) | *Q_B_* | 0*.*016 | (Kandel et al., 2000) |
|  | *Q_H_* | 0*.*083 | (Kandel et al., 2000) (Hayes et al., 2014) |
|  | *Q_A_* | 0*.*008 | (Kandel et al., 2000) (Hayes et al., 2014) |
|  | *Q_L_* | 0*.*021 | (Hayes et al., 2014) |
|  | *Q_G_* | 0*.*042 | (Hayes et al., 2014) |
|  | *Q_K_* | 0*.*016 | (Welch et al., 1995) |
| Parameter type | Parameter | Value ($L$) | Reference |
|  | $V_{B}$ | 5*.*989 × 10^−4^ | (Sahin et al., 2011) |
|  | $V_{H}$ | 3 × 10^−3^ | (Edgren and von, 1973) |
|  | $V_{A}$ | 1*.*9 × 10^−2^ | (Schoeffner, 1999) |
|  | $V_{L}$ | 1*.*192 × 10^−2^ | (McConnell et al., 2008) |
|  | $V_{G}$ | 7*.*8 × 10^−3^ | (Strømmen et al., 2004) |
|  | $V_{K}$ | 1*.*58 × 10^−3^ | (Ellenbogen and Rengachary, 2005) |
| Parameter type | Parameter | Value | Reference |
| Tissue fractional blood volume (f) | $f_{B}$ | 0.1 | (Nieuwenhuys et al., 2014) |
|  | $f_{H}$ | 0.1 | Approximation |
|  | $f_{A}$ | 0.1 | Approximation |
|  | $f_{L}$ | 0.1 | Approximation |
|  | $f_{G}$ | 0.1 | Approximation |
|  | $f_{K}$ | 0.1 | Approximation |
| Parameter type | Parameter | Value (*dm*^2^) | Reference |
| Tissue Surface Area (S) | $S_{B}$ | $6x{10}^{-2}$ | (Permezel and Webling, 1971) |
|  | $S_{H}$ | 1*.*005 × 10^−1^ | Computation |
|  | $S_{A}$ | 3*.*443 × 10^−1^ | Computation |
|  | $S_{L}$ | 2*.*523 × 10^−1^ | Computation |
|  | $S_{G}$ | 2*.*46 | (Permezel and Webling, 1971) |
|  | $S_{K}$ | 8*.*264 × 10^−2^ | Computation |

(e)

| Tissue type | Training dataset size |
| --- | --- |
| Brain | (113 × 1449) |
| Heart | (106 × 1449) |
| Adipose | (100 × 1449) |
| Liver | (115 × 1449) |
| Gut | (60 × 1449) |
| Kidney | (118 × 1449) |

(f)

| Chemical | Substrate | Enzyme | *K_m_* (mol/l) | *Vmax*  (mol/sec) | Reference |
| --- | --- | --- | --- | --- | --- |
| Superoxide | superoxide | superoxide dismutase | 3 × 10^−3^ | 0*.*001375 | (Karadag and Bilgin, 2010) |
| Hydrogen peroxide | Hydrogen peroxide | Catalase peroxide | 0.85 × 10^-3^ | 6.36 × 10^-6^ | (Kengen et al., 2001) |
| Hydrogen peroxide | Xanthine | Xanthine oxidase | 3.38 × 10^-6^ | 2.07 × 10^-6^ | (Bassingthwaighte and Chinn, 2013) |
| Hydrogen peroxide | Sulfite | Sulfite oxidase | 3.34 × 10^−3^ | 1.88 × 10^-5^ | (Ganai et al., 2006) |

Table S3: Predictions using QSPR. (a) Partition coefficient predicted for propionic acid, hydrogen peroxide and SOX. (b) Renal clearance (L/s) predicted for propionic acid, hydrogen peroxide and sulphur dioxide.

(a)

| Tissue type | *K_T_* for propionic acid | *K_T_* for hydrogen perox-  ide | *K_T_* for superoxide |
| --- | --- | --- | --- |
| Brain | 1.703 | 3.056 | 2.79 |
| Heart | 5.269 | 4.935 | 4.915 |
| Adipose | 7.993 | 8.963 | 10.016 |
| Liver | 12.655 | 12.243 | 5.301 |
| Gut | 10.138 | 10.075 | 5.167 |
| Kidney | 9.739 | 8.964 | 4.718 |

(b)

| *CL_R_* for propionic acid | *CL_R_* for hydrogen peroxide | *CL_R_* for superoxide |
| --- | --- | --- |
| 1.45 | 7.3 | 7.3 |

**Ten microbe community model construction**

To add more diversity in the gut microbiota the community model was expanded to ten microbial system. Wherein, we considered *Bacteroides vulgatus, Clostridium perfringens, Clostridium difficile, Ruminococcus torques, Shigella flexneri* bacteria as five harmful bacteria and *Akkermansia muciniphila, Bifidobacterium longum longum, Desulfovibrio desulfuricans subsp, Lactobacillus acidophilus, Prevotella ruminicola* five beneficial bacteria which are expected to intervene and reduce the toxins and restores the intestinal barrier. These ten bacteria is then combined with the small intestine enterocyte model to represent complex gut model (as shown in Table 2).

The simulation results with ten bacterial model are documented in Additional file 9 to 14.

Table S4:

| Ten microbial community: Individual and combined model specification | | | | | | | | | | | | |
| --- | --- | --- | --- | --- | --- | --- | --- | --- | --- | --- | --- | --- |
| Model | Diet | BV | DD | CP | LA | BL | AKK | CLS | PRE | RT | SH | SIEC |
| *Gut microbiome* | *Western* | 4.9256 | 2.2467 | 4.9257 | 3.4168 | 1.4948 | 4.7062 | 3.4168 | 3.4168 | 4.9250 | 4.9257 | 0.0428 |
|  | *High-Fiber* | 4.8946 | 2.5307 | 4.9857 | 3.6148 | 1.5461 | 4.9256 | 3.4168 | 3.5268 | 4.9256 | 4.9257 | 0.0428 |
| *Gut microbiome - Harmful* | *Western* | 4.9856 | - | 5.1223 | - | - | - | 3.8565 | - | 5.2250 | 5.1258 | 0.0928 |
|  | *High-Fiber* | 4.9556 | - | 4.9886 | - | - | - | 3.6168 | - | 5.1256 | 5.0654 | 0.0926 |
| *Gut microbiome - Beneficial* | *Western* | - | 2.0523 | - | 1.4834 | 1.3581 | 4.1360 | - | 3.4168 | - | - | 0.1028 |
|  | *High-Fiber* | - | 2.0897 | - | 1.4834 | 1.3721 | 4.3405 | - | 3.4168 | - | - | 0.1428 |

Table S5:

|  |  | Weights of objective function reactions | | | |
| --- | --- | --- | --- | --- | --- |
| S.No. |  | Beneficial bacteria % | | | |
|  | Objective Reaction | 20% | 40% | 60% | 80% |
| 1 | LA_biomass473 | 0.145455 | 0.109091 | 0.072727 | 0.036364 |
| 2 | BL_biomass318 | 0.036364 | 0.072727 | 0.109091 | 0.145455 |
| 3 | BV_biomass318 | 0.145455 | 0.109091 | 0.072727 | 0.036364 |
| 4 | DD_biomass376 | 0.036364 | 0.072727 | 0.109091 | 0.145455 |
| 5 | CP_biomass012 | 0.036364 | 0.072727 | 0.109091 | 0.145455 |
| 6 | AKK_biomass328 | 0.036364 | 0.072727 | 0.109091 | 0.145455 |
| 7 | CLS_biomass525 | 0.145455 | 0.109091 | 0.072727 | 0.036364 |
| 8 | PRE_biomass525 | 0.036364 | 0.072727 | 0.109091 | 0.145455 |
| 9 | RT_biomass345 | 0.145455 | 0.109091 | 0.072727 | 0.036364 |
| 10 | SH_biomass353 | 0.145455 | 0.109091 | 0.072727 | 0.036364 |
| 11 | biomass_reactionIEC01b | 0.090909 | 0.090909 | 0.090909 | 0.090909 |

Table S6:

| Ten microbial community | | | | | | | | |
| --- | --- | --- | --- | --- | --- | --- | --- | --- |
| Biomass reaction | Beneficial percentage | | | | | | | |
|  | 20% | | 40% | | 60% | | 80% | |
|  | Diet | | | | | | | |
|  | High-fiber | Western | High-fiber | western | High fiber | western | High fiber | Western |
| BV | 1.024714 | 1.032979 | 0.693516 | 0.706753 | 0.317472 | 0.322282 | 0.019241 | 0.024051 |
| DD | 0.0336720 | 0.038482 | 0.3271 | 0.33191 | 0.671469 | 0.684706 | 0.826401 | 0.958625 |
| CP | 0.3216448 | 0.313477 | 0.217686 | 0.214478 | 0.09965 | 0.097803 | 0.006039 | 0.007299 |
| LA | 0.0233571 | 0.026694 | 0.226898 | 0.230235 | 0.465776 | 0.474958 | 0.573247 | 0.664967 |
| BL | 0.0336712 | 0.037831 | 0.327092 | 0.32629 | 0.671454 | 0.673113 | 0.826383 | 0.942394 |
| AKK | 0.0160692 | 0.017553 | 0.156101 | 0.151392 | 0.320444 | 0.312311 | 0.394382 | 0.437253 |
| CLS | 0.7108268 | 0.716559 | 0.48108 | 0.490262 | 0.220225 | 0.223562 | 0.013347 | 0.016684 |
| PRE | 0.0336712 | 0.036767 | 0.327092 | 0.31712 | 0.671454 | 0.654195 | 0.826383 | 0.915908 |
| RT | 0.7108268 | 0.716559 | 0.48108 | 0.490262 | 0.220225 | 0.223562 | 0.013347 | 0.016684 |
| SH | 1.0247224 | 1.032986 | 0.693521 | 0.706758 | 0.317474 | 0.322285 | 0.019241 | 0.024051 |
| sIEC | 0.0039625 | 0.004057 | 0.004385 | 0.004461 | 0.004247 | 0.004323 | 0.002898 | 0.003489 |

Table S7:

| Brain model connected with ten microbial community  ATP production fluxes in brain cells on consumption of Western diet (mmol/gWhr) | |
| --- | --- |
| Brain cell | ATP production |
| Astrocyte | 3.3883 |
| Glutamate neuron | 4.1418 |
| GABA neuron | 4.1437 |

Table S8: Summary of effect of gut bacteria, diet, and reactive species metabolites on the metabolism of gut and brain. Only top ten pathways are shown. (a) Metabolic pathways affected in the gut by addition of beneficial bacteria and changing the diet from western to high-fiber. (b) Metabolic pathways affected in the gut and brain due to increased oxidative stress.

*(a)*

| S.No. | Pathways affected by addition of beneficial bacteria to ’purely’ autistic gut | Pathways affected by change in diet |
| --- | --- | --- |
| 1 | Purine catabolism | Purine catabolism |
| 2 | Nucleotide interconversion | Glycolysis/gluconeogenesis |
| 3 | Glycolysis/gluconeogenesis | Fructose and mannose metabolism |
| 4 | Citric acid cycle | Citric acid cycle |
| 5 | Valine, leucine, and isoleucine metabolism | Pyruvate metabolism |
| 6 | Propanoate metabolism | Nucleotide interconversion |
| 7 | Pyruvate metabolism | NAD metabolism |
| 8 | Vitamin B6 metabolism | Purine synthesis |
| 9 | NAD metabolism | Vitamin B6 metabolism |
| 10 | Purine synthesis | Pyrimidine catabolism |

*(b)*

| S.No. | Pathways affected in the gut | Pathways affected in the brain |
| --- | --- | --- |
| 1 | Purine catabolism | ROS Detoxification |
| 2 | Citric acid cycle | Oxidative Phosphorylation |
| 3 | Alanine and aspartate metabolism | Pyruvate Metabolism |
| 4 | Purine synthesis | Glutamate metabolism |
| 5 | Vitamin B6 metabolism | NAD Metabolism |
| 6 | NAD metabolism | Citric Acid Cycle |
| 7 | Pyrimidine catabolism | Urea cycle/amino group metabolism |
| 8 | Glycolysis/gluconeogenesis | Alanine and Aspartate Metabolism |
| 9 | Arginine and Proline Metabolism | Valine, Leucine, and Isoleucine Metabolism |
| 10 | Nucleotide interconversion | Folate Metabolism |

Table S9: Summary of the effect of beneficial bacteria and diet on the secretion products of the harmful bacteria. Only top 15 bacterial secretion products are shown. (a) Harmful bacterial secretion products (b) Harmful bacterial secretion products that are affected by addition of beneficial bacteria, c) The change observed upon addition of the corresponding beneficial bacteria, (d) Increased secretion products after changing the diet from western to high-fiber.

| **Ten microbial community** | | | | |
| --- | --- | --- | --- | --- |
| **Rank** | **a) Harmful Bacteria Products** | **b) Beneficial Bacteria Products** | **c) Common** | **d) Effect of diet change** |
| 1 | Formate | Formate | Carbon dioxide | Hydrogen |
| 2 | Hydrogen | Adenylate | Hydrogen | acetate |
| 3 | Acetate | Acetate | Oxygen | Carbon dioxide |
| 4 | Ammonium | Propionate | Uric acid | proton |
| 5 | Guanine | Carbon Dioxide | lactate | acetaldehyde |
| 6 | Carbon Dioxide | Oxygen | Priponic acid | lactate |
| 7 | Hydrogen ions | Uric acid | alanine | Propionate |
| 8 | Adenine | Lactate | Glycine | pyruvate |
| 9 | Succinate | Propionic acid | Niacinamide | L-serine |
| 10 | Propionate | Alanine | Nicotinic acid | L-alanine |
| 11 | Lactate | Hydrogen | Nitric acid | Ammonium |
| 12 | Ethanol | Glycine | L-serine | Formate |
| 13 | Amino acetaldehyde | Niacinamide | L-Cysteine | 2-Oxobutanoate |
| 14 | Pyruvate | Nicotinic acid | L-Threonine | Succinate |
| 15 | Malate | Nitric oxide | Water | Propane-1,3-diol |

Table S10: Models compared and the number of reactions affected. Default diet is western diet if not mentioned otherwise.

| Effect | 1^st^ Model of comparison | 2^nd^ Model of comparison | Reactions compared (total number) | No. of reactions affected or shifted |
| --- | --- | --- | --- | --- |
| Effect of harmful bacteria | SIEC | Gut microbiome  harmful | SIEC (1295) | 620 |
| Effect of beneficial bacteria | Gut microbiome  harmful | Gut microbiome | Harmful bacterial secretion products (268) | 73 |
| Effect of diet | Gut microbiome  harmful | Gut microbiome | SIEC (1295) | 627 |
|  | Gut microbiome-Western diet | Gut microbiome-High fiber diet | Bacterial secretion products (268) | 84 |
|  | Gut microbiome-Western diet | Gut microbiome-High fiber diet | SIEC (1295) | 681 |
| Effect of oxidative Stress | Gut microbiome–toxic | Gut microbiome – non toxic | SIEC (1295) | 578 |
|  | Brain-toxic | Brain-non toxic | Brain (1542) | 1048 |

Table S11. Individual and combined model Specifications

| Bacteria | Number of reactions | Number of metabolites | References |
| --- | --- | --- | --- |
| Individual model specification | | | |
| Bacteroides-vulgatus-ATCC-8482 | 1302 | 1110 | (Magnúsdóttir, S., Heinken, A., Kutt, L., Ravcheev, D. A., Bauer, E., Noronha, A., et al. 2016) |
| Desulfovibrio-desulfuricans-subsp-desulfuricans-DSM-642 | 1279 | 1148 | (Magnúsdóttir, S., Heinken, A., Kutt, L., Ravcheev, D. A., Bauer, E., Noronha, A., et al. 2016) |
| Clostridium-perfringens-ATCC-13124 | 1307 | 1115 | (Magnúsdóttir, S., Heinken, A., Kutt, L., Ravcheev, D. A., Bauer, E., Noronha, A., et al. 2016) |
| Lactobacillus-acidophilus-ATCC-4796 | 756 | 728 | (Magnúsdóttir, S., Heinken, A., Kutt, L., Ravcheev, D. A., Bauer, E., Noronha, A., et al. 2016) |
| Bifidobacterium-longum-longum-JDM301 | 1054 | 959 | (Magnúsdóttir, S., Heinken, A., Kutt, L., Ravcheev, D. A., Bauer, E., Noronha, A., et al. 2016) |
| Small Intestine Enetrocyte Cell model | 1282 | 844 | (Sahoo, S., and Thiele, I. 2013) |
| GABAergic neuronal model | 1068 | 993 | (Lewis, N. E., Schramm, G., Bordbar, A., Schellenberger, J., Andersen, M. P., Cheng, J. K., et al. 2010) |
| Glutamatergic neuronal model | 1067 | 993 | (Lewis, N. E., Schramm, G., Bordbar, A., Schellenberger, J., Andersen, M. P., Cheng, J. K., et al. 2010) |
| Combined model specification | | | |
| Gut microbiome – Beneficial | 3177 | 2611 | - |
| Gut microbiome – Harmful | 5323 | 4365 | - |
| Gut microbiome | 7137 | 6056 | - |
| Brain | 1542 | 1428 | - |

B.

| Ten bacterial community | | | |
| --- | --- | --- | --- |
| Bacteria | Number of reactions | Number of metabolites | References |
| Individual model specification | | | |
| Bacteroides-vulgatus-ATCC-8482 | 1302 | 1110 | (Magnúsdóttir, S., Heinken, A., Kutt, L., Ravcheev, D. A., Bauer, E., Noronha, A., et al. 2016) |
| Desulfovibrio-desulfuricans-subsp-desulfuricans-DSM-642 | 1279 | 1148 | (Magnúsdóttir, S., Heinken, A., Kutt, L., Ravcheev, D. A., Bauer, E., Noronha, A., et al. 2016) |
| Clostridium-perfringens-ATCC-13124 | 1307 | 1115 | (Magnúsdóttir, S., Heinken, A., Kutt, L., Ravcheev, D. A., Bauer, E., Noronha, A., et al. 2016) |
| Lactobacillus-acidophilus-ATCC-4796 | 756 | 728 | (Magnúsdóttir, S., Heinken, A., Kutt, L., Ravcheev, D. A., Bauer, E., Noronha, A., et al. 2016) |
| Bifidobacterium-longum-longum-JDM301 | 1054 | 959 | (Magnúsdóttir, S., Heinken, A., Kutt, L., Ravcheev, D. A., Bauer, E., Noronha, A., et al. 2016) |
| Akkermansia_muciniphila_ATCC_BAA_835 | 1142 | 994 | (Magnúsdóttir, S., Heinken, A., Kutt, L., Ravcheev, D. A., Bauer, E., Noronha, A., et al. 2016) |
| Clostridium_difficile_NAP07 | 1297 | 1123 | (Magnúsdóttir, S., Heinken, A., Kutt, L., Ravcheev, D. A., Bauer, E., Noronha, A., et al. 2016) |
| Prevotella_ruminicola_23 | 1146 | 1017 | (Magnúsdóttir, S., Heinken, A., Kutt, L., Ravcheev, D. A., Bauer, E., Noronha, A., et al. 2016) |
| Ruminococcus_torques_ATCC_27756 | 995 | 901 | (Magnúsdóttir, S., Heinken, A., Kutt, L., Ravcheev, D. A., Bauer, E., Noronha, A., et al. 2016) |
| Shigella_flexneri_2002017 | 1756 | 1381 | (Magnúsdóttir, S., Heinken, A., Kutt, L., Ravcheev, D. A., Bauer, E., Noronha, A., et al. 2016) |
| Small Intestine Enetrocyte Cell model | 1282 | 844 | (Sahoo, S., and Thiele, I. 2013) |
| GABAergic neuronal model | 1068 | 993 | (Lewis, N. E., Schramm, G., Bordbar, A., Schellenberger, J., Andersen, M. P., Cheng, J. K., et al. 2010) |
| Glutamatergic neuronal model | 1067 | 993 | (Lewis, N. E., Schramm, G., Bordbar, A., Schellenberger, J., Andersen, M. P., Cheng, J. K., et al. 2010) |
| Combined model specification | | | |
| Gut microbiome – Beneficial | 3177 | 2611 | - |
| Gut microbiome – Harmful | 5323 | 4365 | - |
| Gut microbiome | 7137 | 6056 | - |
| Brain | 1542 | 1428 | - |

Table S12.

(a)

| Reaction name | Reaction abbreviation | Reaction formula |
| --- | --- | --- |
| Exchange/demand reaction | EX_ppa(u) | ppa[u] <=> |
| Transport, extracellular | O2St | $o_{2}s$[c] <=> $o_{2}s$[e] |
| Transport, mitochondrial | O2Stm | $o_{2}s$[c] <=> $o_{2}s$[m] |
| Transport, extracellular | H2O2te | $h_{2}o_{2}$[c] -> $h_{2}o_{2}$[e] |
| Exchange/demand reaction | PPA_output | $\mathrm{ppa}$[e] -> |
| Exchange/demand reaction | PPA_input | -> $\mathrm{ppa}$[e] |
| Exchange/demand reaction | h2o2_output | $h_{2}o_{2}$[e] -> |
| Exchange/demand reaction | h2o2_input | -> $h_{2}o_{2}$[e] |
| Exchange/demand reaction | o2s_output | $o_{2}s$[e] -> |
| Exchange/demand reaction | o2s_input | -> $o_{2}s$[e] |

(b)

| Metabolite name | Metabolite abbreviation | Metabolite formula |
| --- | --- | --- |
| Propionate (n-C3:0) | ppa[e] | $C_{3}H_{5}O_{2}$ |
| Propionate (n-C3:0) | ppa[c] | $C_{3}H_{5}O_{2}$ |
| Propionate (n-C3:0) | ppa[m] | $C_{3}H_{5}O_{2}$ |
| AMP | amp[m] | $C_{10}H_{12}N_{5}O_{7}P$ |
| Hydrogen peroxide | h2o2[e] | $H_{2}O_{2}$ |
| Superoxide anion | o2s[c] | $O_{2}$ |
| Superoxide anion | o2s[e] | $O_{2}$ |

Table S13: Production and consumption reaction of toxins

| Reaction name | Reaction formula |
| --- | --- |
| $\mathbf{H}_{\mathbf{2}}\mathbf{O}_{\mathbf{2}}$output: | $H_{2}O_{2}$ [e] -> |
| $\mathbf{H}_{\mathbf{2}}\mathbf{O}_{\mathbf{2}}$ input: | -> $H_{2}O_{2}$ [e] |
| $\mathbf{O}_{\mathbf{2}}\mathbf{S}$ output: | $O_{2}S$ [e] -> |
| $\mathbf{O}_{\mathbf{2}}\mathbf{S}$ input: | -> $O_{2}S$ [e] |

Table S14: Production and consumption reaction of toxins

| Reaction name | Reaction formula |
| --- | --- |
| $\mathbf{H}_{\mathbf{2}}\mathbf{O}_{\mathbf{2}}$output: | $H_{2}O_{2}$ [e] -> |
| $\mathbf{H}_{\mathbf{2}}\mathbf{O}_{\mathbf{2}}$ input: | -> $H_{2}O_{2}$ [e] |
| $\mathbf{O}_{\mathbf{2}}\mathbf{S}$ output: | $O_{2}S$ [e] -> |
| $\mathbf{O}_{\mathbf{2}}\mathbf{S}$ input: | -> $O_{2}S$ [e] |

Table S15: Objective function reaction in different models

| Model | Net objective | Objective function reactions | | |
| --- | --- | --- | --- | --- |
| Gut microbiome | Toxin production | $H_{2}O_{2}\mathrm{output}$ | $O_{2}S output$ |  |
| Gut microbiome | Toxin consumption | $H_{2}O_{2}\mathrm{input}$ | $O_{2}S input$ |  |
| Brain | ATP production | $DM atp\left( c \right)\mathrm{GluN}$ | $DM atp(c)$ | $DM atp\left( c \right)\mathrm{GabaN}$ |
| Brain | Toxin production | $H_{2}O_{2}\mathrm{output}$ | $O_{2}S output$ |  |
| Brain | Toxin consumption | $H_{2}O_{2}\mathrm{input}$ | $O_{2}S input$ |  |
| Brain | Neurotransmitter production | $DM 4abut[cGabaN]$ | $DM glu L[cGluN]$ |  |

Table S16: ATP production fluxes in brain cells on consumption of Western diet (mmol/gDW/hr)

| Brain model connected with five microbial community  ATP production fluxes in brain cells on consumption of Western diet (mmol/gWhr) | |
| --- | --- |
| Brain cell | ATP production |
| Astrocyte | 3.3851 |
| Glutamate neuron | 4.1379 |
| GABA neuron | 4.1398 |

Table S17: Top 15 bacterial secretion products for the harmful and beneficial bacteria

| S.No. | Harmful Bacteria | Beneficial bacteria |
| --- | --- | --- |
| 1 | Formate | Acetate |
| 2 | Hydrogen | Hydrogen ions |
| 3 | Acetate | Aminoacetaldehyde |
| 4 | Ammonium | Lactate |
| 5 | Guanine | Formate |
| 6 | Carbon Dioxide | Ethanol |
| 7 | Hydrogen ions | Carbon Dioxide |
| 8 | Adenine | Succinate |
| 9 | Succinate | Ammonium |
| 10 | Propionate | L-alanine |
| 11 | Lactate | L-serine |
| 12 | Ethanol | L-threonine |
| 13 | Aminoacetaldehyde | L-glutamine |
| 14 | Pyruvate | L-asparagine |
| 15 | Malate | Xanthine |

Table S18: (a) Top 10 gut metabolism pathways affected by harmful bacteria, and subsequently by adding beneficial bacteria; and affected by change in diet (b) Effect of oxidative stress on the gut and brain

(a)

| S.No. | Pathways affected by addition of beneficial bacteria to ’purely’ autistic gut | Pathways affected by change in diet |
| --- | --- | --- |
| 1 | Purine catabolism | Purine catabolism |
| 2 | Nucleotide interconversion | Glycolysis/gluconeogenesis |
| 3 | Glycolysis/gluconeogenesis | Fructose and mannose metabolism |
| 4 | Citric acid cycle | Citric acid cycle |
| 5 | Valine, leucine, and isoleucine metabolism | Pyruvate metabolism |
| 6 | Propanoate metabolism | Nucleotide interconversion |
| 7 | Pyruvate metabolism | NAD metabolism |
| 8 | Vitamin B6 metabolism | Purine synthesis |
| 9 | NAD metabolism | Vitamin B6 metabolism |
| 10 | Purine synthesis | Pyrimidine catabolism |

(b)

| S.No. | Pathways affected in the gut | Pathways affected in the brain |
| --- | --- | --- |
| 1 | Purine catabolism | ROS Detoxification |
| 2 | Citric acid cycle | Oxidative Phosphorylation |
| 3 | Alanine and aspartate metabolism | Pyruvate Metabolism |
| 4 | Purine synthesis | Glutamate metabolism |
| 5 | Vitamin B6 metabolism | NAD Metabolism |
| 6 | NAD metabolism | Citric Acid Cycle |
| 7 | Pyrimidine catabolism | Urea cycle/amino group metabolism |
| 8 | Glycolysis/gluconeogenesis | Alanine and Aspartate Metabolism |
| 9 | Arginine and Proline Metabolism | Valine, Leucine, and Isoleucine Metabolism |
| 10 | Nucleotide interconversion | Folate Metabolism |

**References**

Balmus, I., Ciobica, A., Trifan, A., and Stanciu, C. 2016. "The implications of oxidative stress and antioxidant therapies in Inflammatory Bowel Disease: Clinical aspects and animal models." *Saudi J Gastroenterol* 22 (3). doi:10.4103/1319-3767.173753.

Bassingthwaighte, J. B., and Chinn, T. M. 2013. "Reexamining Michaelis-Menten enzyme kinetics for xanthine oxidase." *Advances in Physiology Education* 37: 37–48. doi:10.1152/advan.00107.2012.

Bhattacharyya, A., Chattopadhyay, R., Mitra, S., and Crowe, S. E. 2014. "Oxidative Stress: An Essential Factor in the Pathogenesis of Gastrointestinal Mucosal Diseases." *Physiological Reviews* 94: 329-354. doi:10.1152/physrev.00040.2012.

Chauhan, A., and Chauhan, V. 2006. "Oxidative stress in autism." *Pathophysiology* 13: 171–181. doi:10.1016/j.pathophys.2006.05.007.

Coghlan, S., Horder, J., Inkster, B., Mendez, M. A., Murphy, D. G., and Nutt, D. J. 2012. "GABA system dysfunction in autism and related disorders: From synapse to symptoms." *Neuroscience & Biobehavioral Reviews* 36: 2044–2055. doi:10.1016/j.neubiorev.2012.

de Theije, C. G. M., Wu, J., da Silva, S. L., Kamphuis, P. J., Garssen, J., Korte, S. M., et al. 2011. "Pathways underlying the gut-to-brain connection in autism spectrum disorders as future targets for disease management." *European Journal of Pharmacology* 668: S70-S80. doi:10.1016/j.ejphar.2011.07.013.

Downs, R., Perna, J., Vitelli, A., Cook, D., and Dhurjati, P. 2014. "Model-based hypothesis of gut microbe populations and gut/brain barrier permeabilities in the development of regressive autism." *Medical Hypotheses* 83: 649-655. doi:10.1016/j.mehy.2014.09.005.

Edgren, J., and von Knorring, J. 1973. "Radiological determination of heart volume in rats." *Experientia* 29: 1174–1176. doi:10.1007/bf01946791.

Ellenbogen RG, Rengachary SS. 2005. *Principles of neurosurgery. .* New York: Elsevier Mosby.

Finegold, S. M. 2011. "Desulfovibrio species are potentially important in regressive autism." *Medical Hypotheses* 77(2): 270–274. doi:10.1016/j.mehy.2011.04.032.

Finegold, S. M. 2008. "Therapy and epidemiology of autism–clostridial spores as key elements." *Medical Hypotheses* 70: 508–511. doi:10.1016/j.mehy.2007.07.019.

Finegold, S. M., Dowd, S. E., Gontcharova, V., Liu, C., Henley, K. E., Wolcott, R. D., et al. 2010. "Pyrosequencing study of fecal microflora of autistic and control children." *Anaerobe* 16: 444–453. doi:10.1016/j.anaerobe.2010.06.008.

Ganai, B. A., A. Masood, M. A. Zargar, and M. B. Syed. 2006. "Kinetics of sulfite oxidase purified from Malva sylvestris." *J Ind Pollut Contr* 22: 77-82.

Graham, H., Walker, M., Jones, O., Yates, J., Galetin, A., and Aarons, L. 2011. "Comparison of in-vivo and in-silico methods used for prediction of tissue: plasma partition coefficients in rat." *Journal of Pharmacy and Pharmacology* 64: 383–396. doi:10.1111/j.2042-7158.2011.01429.x.

Hayes, A. Wallace, and Claire L. Kruger, eds. . 2014. *Hayes' principles and methods of toxicology. .* Crc Press.

Heberling, C. A., Dhurjati, P. S., and Sasser, M. 2013. "Hypothesis for a systems connectivity model of autism spectrum disorder pathogenesis: Links to gut bacteria, oxidative stress, and intestinal permeability." *Medical Hypotheses* 80: 264–270. doi:10.1016/j.mehy.2012.11.044.

James, S. J., Cutler, P., Melnyk, S., Jernigan, S., Janak, L., Gaylor, D. W., et al. 2004. "Metabolic biomarkers of increased oxidative stress and impaired methylation capacity in children with autism." *The American Journal of Clinical Nutrition* 80: 1611–1617. doi:10.1093/ajcn/80.6.1611.

James, S. J., Cutler, P., Melnyk, S., Jernigan, S., Janak, L., Gaylor, D. W., et al. 2004. "Metabolic biomarkers of increased oxidative stress and impaired methylation capacity in children with autism." *The American Journal of Clinical Nutrition* 80: 1611–1617. doi:10.1093/ajcn/80.6.1611.

Jansson, R., Bredberg, U., and Ashton, M. 2008. "Prediction of Drug Tissue to Plasma Concentration Ratios Using a Measured Volume of Distribution in Combination With Lipophilicity." *Journal of Pharmaceutical Sciences* 97: 2324–2339. doi:10.1002/jps.21130.

Jones, R. D., Jones, H. M., Rowland, M., Gibson, C. R., Yates, J. W. T., Chien, J. Y., et al. 2011. "PhRMA CPCDC initiative on predictive models of human pharmacokinetics, part 2: Comparative assessment of prediction methods of human volume of distribution." *Journal of Pharmaceutical Sciences* 100: 4074-4089. doi:10.1002/jps.22553.

Kandel, Eric R., James H. Schwartz, Thomas M. Jessell, Steven Siegelbaum, A. James Hudspeth, and Sarah Mack, eds. 2000. *Principles of neural science.* Vol. 4. New York: McGraw-hill.

Kang, D., Adams, J.B., Gregory, A.C. 2017. "Microbiota Transfer Therapy alters gut ecosystem and improves gastrointestinal and autism symptoms: an open-label study." *Microbiome* 5: 10. doi:https://doi.org/10.1186/s40168-016-0225-7.

Karadag, H., and Bilgin, R. 2010. "Purification of Copper-Zinc Superoxide Dismutase from Human Erythrocytes and Partial Characterization." *Biotechnology & Biotechnological Equipment* 24: 1653–1656. doi:10.2478/v10133-010-0021-7.

Katritzky, A. R., Kuanar, M., Fara, D. C., Karelson, M., Acree, W. E., Jr., Solov’ev, V. P., et al. 2005. "QSAR modeling of blood:air and tissue:air partition coefficients using theoretical descriptors." *Bioorganic & Medicinal Chemistry* 13: 6450–6463. doi:10.1016/j.bmc.2005.06.066.

Kengen, S., Bikker, F., Hagen, W., Vos, W., and Oost, J. 2001. "Characterization of a catalase-peroxidase from the hyperthermophilic archaeon Archaeoglobus fulgidus." *Extremophiles* 5: 323–332. doi:10.1007/s007920100208.

Kondoh, H., et al. 2007. "Protection from oxidative stress by enhanced glycolysis; a possible mechanism of cellular immortalization." *Histology and histopathology* 22 (1): 85-90.

Kurochkin, I., Khrameeva, E., Tkachev, A., Stepanova, V., Vanyushkina, A., Stekolshchikova, E., Li, Q., Zubkov, D., Shichkova, P., Halene, T., Willmitzer, L., Giavalisco, P., Akbarian, S., & Khaitovich, P. n.d. "Metabolome signature of autism in the human prefrontal cortex." *Communications biology.* doi:10.1038/s42003-019-0485-4.

Lam, G., Chen, M.-L., and Chiou, W. L. 1982. "Determination of Tissue to Blood Partition Coefficients in Physiologically-Based Pharmacokinetic Studies." *Journal of Pharmaceutical Sciences* 71: 454-456. doi:10.1002/jps.2600710421.

Liu, H. X., Yao, X. J., Zhang, R. S., Liu, M. C., Hu, Z. D., and Fan, B. T. 2005. "Prediction of the tissue/blood partition coefficients of organic compounds based on the molecular structure using least-squares support vector machines." *J Comput Aided Mol Des* 19: 499-508. doi:10.1007/s10822-005-9003-5.

MacFabe, D. F. 2012. "Short-chain fatty acid fermentation products of the gut microbiome: implications in autism spectrum disorders." *Microbial Ecology in Health & Disease* 23. doi:10.3402/mehd.v23i0.19260.

MACFABE, D., CAIN, D., RODRIGUEZCAPOTE, K., FRANKLIN, A., HOFFMAN, J., BOON, F., et al. 2007. "Neurobiological effects of intraventricular propionic acid in rats: Possible role of short chain fatty acids on the pathogenesis and characteristics of autism spectrum disorders." *Behavioural Brain Research* 176: 146-169. doi:0.1016/j.bbr.2006.07.025.

McConnell, E. L., Basit, A. W., and Murdan, S. 2008. "Measurements of rat and mouse gastrointestinal pH, fluid and lymphoid tissue, and implications for in-vivo experiments." *Journal of Pharmacy and Pharmacology* 60: 63–70. doi:10.1211/jpp.60.1.0008.

Newton, Cummings, Macfarlane, and Macfarlane. 1998. "Growth of a human intestinal Desulfovibrio desulfuricans in continuous cultures containing defined populations of saccharolytic and amino acid fermenting bacteria." *J Appl Microbiol* 85: 372–380. doi:10.1046/j.1365-2672.1998.00522.x.

Nieuwenhuys, Rudolf, J. Hans, and Charles Nicholson. . 2014. *The central nervous system of vertebrates. .* Springer.

Orth, J. D., Thiele, I., and Palsson, B. Ø. 2010. "What is flux balance analysis?" *Nat Biotechnol* 245–248. doi:10.1038/nbt.1614.

Permezel, N. C., and D. D. Webling. . n.d. "The length and mucosal surface area of the small and large gut in young rats ." *Journal of anatomy* 108.

Rodgers, T., and Rowland, M. 2006. "Physiologically based pharmacokinetic modelling 2: Predicting the tissue distribution of acids, very weak bases, neutrals and zwitterions." *Journal of Pharmaceutical Sciences* 95: 1238–1257. doi:10.1002/jps.20502.

Rodgers, T., Leahy, D., and Rowland, M. 2005. "Physiologically Based Pharmacokinetic Modeling 1: Predicting the Tissue Distribution of Moderate-to-Strong Bases." *Journal of Pharmaceutical Sciences* 94: 1259–1276. doi:10.1002/jps.20322.

Rossignol, D., Frye, R. 2012. "Mitochondrial dysfunction in autism spectrum disorders: a systematic review and meta-analysis. ." *Mol Psychiatry* 290–314 . doi:10.1038/mp.2010.136.

Sahin, B., Aslan, H., Unal, B., Canan, S., Bilgic, S., Kaplan, S., et al. 2011. "BRAIN VOLUMES OF THE LAMB, RAT AND BIRD DO NOT SHOW HEMISPHERIC ASYMMETRY: A STEREOLOGICAL STUDY." *Image Anal Stereol* 20 (9). doi:10.5566/ias.v20.p9-13.

Sandler, R. H., Finegold, S. M., Bolte, E. R., Buchanan, C. P., Maxwell, A. P., Väisänen, M.-L., et al. 2001. "Short-Term Benefit From Oral Vancomycin Treatment of Regressive-Onset Autism." *J Child Neurol* 15: 429–435. doi:10.1177/088307380001500701.

Schellenberger, J., Que, R., Fleming, R. M. T., Thiele, I., Orth, J. D., Feist, A. M., et al. 2011. "Quantitative prediction of cellular metabolism with constraint-based models: the COBRA Toolbox v2.0." *Nat Protoc* 6: 1290–1307. doi:10.1038/nprot.2011.308.

Schoeffner, D. J. 1999. "ORGAN WEIGHTS AND FAT VOLUME IN RATS AS A FUNCTION OF STRAIN AND AGE." *Journal of Toxicology and Environmental Health, Part A* 56: 449-462. doi:10.1080/009841099157917.

Slavin, Joanne. 2013. "Fiber and Prebiotics: Mechanisms and Health Benefits." *Nutrients* 5 (4): 1417-1435. doi:10.3390/nu5041417.

Strømmen, K., Stormark, T. A., Iversen, B. M., and Matre, K. 2004. "Volume estimation of small phantoms and rat kidneys using three-dimensional ultrasonography and a position sensor." *Ultrasound in Medicine & Biology* 30: 1109–1117. doi:10.1016/j.ultrasmed.

Tibshirani, R. 1996. "Regression Shrinkage and Selection Via the Lasso." *Journal of the Royal Statistical Society: Series B (Methodological)* 58: 267-288. doi:10.1111/j.2517-6161.1996.tb02080.x.

Varma, M. V. S., Feng, B., Obach, R. S., Troutman, M. D., Chupka, J., Miller, H. R., et al. 2009. "Physicochemical Determinants of Human Renal Clearance." *J. Med. Chem.* 52: 4844–4852. doi:10.1021/jm900403j.

Welch, W. J., Deng, X., Snellen, H., and Wilcox, C. S. 1995. "Validation of miniature ultrasonic transit-time flow probes for measurement of renal blood flow in rats." *American Journal of Physiology-Renal Physiology* 268: F175–F178. doi:10.1152/ajprenal.19.

Weston, B., Fogal, B., Cook, D., and Dhurjati, P. 2015. "An agent-based modeling framework for evaluating hypotheses on risks for developing autism: Effects of the gut microbial environment." *Medical Hypotheses* 84: 395–401. doi:10.1016/j.mehy.2015.01.027.

Yap, C. W. 2010. "PaDEL-descriptor: An open source software to calculate molecular descriptors and fingerprints." *J. Comput. Chem.* 32: 1466–1474. doi:10.1002/jcc.21707.

Yun, Y. E., and Edginton, A. N. 2013. "Correlation-based prediction of tissue-to-plasma partition coefficients using readily available input parameters." *Xenobiotica* 43: 839–852. doi:10.3109/00498254.2013.770182.
